# Evolution of two-component quorum sensing systems

**DOI:** 10.1099/acmi.0.000303

**Published:** 2022-01-12

**Authors:** Marina Giannakara, Vassiliki Lila Koumandou

**Affiliations:** ^1^​ Genetics Laboratory, Department of Biotechnology, Agricultural University of Athens, Athens, Greece

**Keywords:** quorum sensing, two component systems, histidine kinase, response regulator, conserved domains, molecular evolution, phylogenetics

## Abstract

Quorum sensing (QS) is a cell-to-cell communication system that enables bacteria to coordinate their gene expression depending on their population density, via the detection of small molecules called autoinducers. In this way bacteria can act collectively to initiate processes like bioluminescence, virulence and biofilm formation. Autoinducers are detected by receptors, some of which are part of two-component signal transduction systems (TCS), which comprise of a (usually membrane-bound) sensor histidine kinase (HK) and a cognate response regulator (RR). Different QS systems are used by different bacterial taxa, and their relative evolutionary relationships have not been extensively studied. To address this, we used the Kyoto Encyclopedia of Genes and Genomes (KEGG) database to identify all the QS HKs and RRs that are part of TCSs and examined their conservation across microbial taxa. We compared the combinations of the highly conserved domains in the different families of receptors and response regulators using the Simple Modular Architecture Research Tool (SMART) and KEGG databases, and we also carried out phylogenetic analyses for each family, and all families together. The distribution of the different QS systems across taxa, indicates flexibility in HK–RR pairing and highlights the need for further study of the most abundant systems. For both the QS receptors and the response regulators, our results indicate close evolutionary relationships between certain families, highlighting a common evolutionary history which can inform future applications, such as the design of novel inhibitors for pathogenic QS systems.

## Data Summary

Please find the supplementary material here: https://doi.org/10.6084/m9.figshare.16732516.v1 [1].

## Introduction

### The mechanism and importance of quorum sensing

Quorum sensing (QS) is a mechanism responsible for regulating a variety of group behaviours and interactions in bacteria, including pathogenicity, biofilm formation, mobility, sporulation, conjugal plasmid transfer, bioluminescence, resistance to antibiotics and production of antibiotics [2, 3]. Apart from affecting bacterial functions and interactions, QS signalling molecules can also interact with host cell receptors, e.g. affecting mouse myoblasts [4] and the central nervous system signalling and behaviour in mice [5]. Despite differences in their molecular mechanisms and regulatory components, all QS systems regulate their target genes in a population-density dependent manner. An extracellular molecule [autoinducer (AI)] is secreted by a group of bacteria and when the number of the bacteria rises, so does the number of these molecules produced in the surrounding area; when the concentration of the AIs overcomes a specific threshold, they bind to either a transmembrane or cytoplasmic receptor, which activates a signal transduction cascade, altering the gene expression of the cells across the population [3].

The quorum sensing mechanism differs between Gram-positive and Gram-negative bacteria. Gram-positive bacteria use modified oligopeptides as AIs [autoinducing peptides (AIPs)]; these bind to transmembrane histidine kinase (HK) receptors, leading to phosphorylation of the HK protein and its cognate response regulator (RR), which then regulates the target genes. The AIPs can also be transported into the cell and interact with cytoplasmic AIP receptors, which act as transcription regulators [3, 6]. Gram-negative bacteria use acyl-homoserine-lactones (AHLs) or *S*-adenosyl methionine (SAM)-products as AIs [7], or 4,5-dihydroxy-2,3-pentanedione (DPD)-derived molecules, collectively known as AI-2 [2]. 3,5-Dimethylpyrazin-2-ol (DPO) is another type of QS signalling molecule for Gram-negative bacteria [8]. In addition, bacteria-derived pyrazinones have recently been reported to constitute the group of AI-3 [9]. Those small molecules can either diffuse through the cell membrane and bind to LuxR-type cytoplasmic receptors, which mediate transcription regulation, or they are detected by transmembrane HKs in a process similar to that of Gram-positive bacteria [6]. Other molecules sensed by the QS receptors include pheromones, adrenaline and noradrenaline, sulphate and phosphate ions [10], fucose [11] and quinolones [5, 12].

Since quorum sensing affects many bacterial processes, its study can have numerous applications. QS inhibition [quorum quenching (QQ)] can be applied to fields such as hospital infections, given the increasing spread of antibiotic-resistant bacteria, phytopathogen control in agriculture and for producing new preservatives in the food industry [13, 14]. The gene products involved in QS mechanisms are consequently possible targets in new antimicrobial strategies and the investigation of their evolutionary relationships can contribute in that direction [15]. It has been suggested that targeting QS imposes less selective pressure, since it does not kill bacteria but only hinders the production of virulence factors [16]. Current research on novel molecules inhibiting QS is characterised by a variety of approaches, e.g. there have been reports of β-turn mimetic-based peptides [17], bacterial lactonases [18] and symbiont-derived molecules [19] as biofilm and virulence inhibitors. Products of plant origin have also demonstrated anti-QS activity: pomegranate rind, which is rich in tannins, can inhibit biofilm formation and motility of *Escherichia coli *, being a potential means of reducing *E. coli* contamination in the food industry [20].

### Two-component system types

The HKs that contribute to QS are part of the two-component signal transduction systems (TCSs). TCS is the main mechanism in bacteria for responding to environmental stimuli and prevails across the entire bacterial kingdom [21]. Although there is a wide variety in TCS mechanisms, they all fall into two main categories: The canonical and the multi-step TCS.

The canonical TCS (His–Asp) comprises two proteins: a transmembrane histidine kinase (HK) and a response regulator (RR) [22]. The HK is a dimeric protein which includes three domains: the sensor, the histidine kinase domain or dimerisation domain (HisKA) and the ATP kinase binding domain (HATPase). The sensor domain binds to the AI and this interaction results in an ATP-dependent autophosphorylation of the conserved histidine of HisKA [22, 23]. The phosphoryl group is then transferred to a protein called response regulator (RR). The RR contains the response regulator domain or receiver domain (REC) in the N-terminal region and the effector domain in the C-terminus [22, 23]. The REC domain includes the conserved aspartate residue, which is phosphorylated; this leads to conformational changes in the structure of the RR [24], which is responsible for controlling quorum sensing-associated behaviours through the effector domain either via protein–protein interactions or by binding to DNA for gene regulation [25].

The multi-step TCS mechanism (His–Asp–Asp) usually includes a hybrid HK (HHK), a histidine phosphotransferase (HPt) and its cognate RR [22]. The structure of the HHK is similar to that of a HK but additionally includes a C-terminal REC domain with a conserved Asp residue. The binding of the signalling molecule induces the autophosphorylation of the His of the HisKA domain and then the phosphoryl group is transferred intramolecularly to the Asp residue of the HHK. Then the phosphotransfer continues to the HPt protein and from there to the RR [21, 22].

### Evolution of TCS

It is suggested that the more variable the environment, the higher the number of the TCS of the organisms living in it. The genes of the HKs and their cognate RRs are usually found on the same operon [21]. The various TCS pathways are the result of lateral gene transfer and/or duplication [26]. The duplication can happen to all of the genes of the operon or to one of them; in the second case, the resulting TCS pathway consists of more than one HK and responds to more than one extracellular signal, or it includes more than one RR and gives multiple responses for a specific signal [21]. Also, due to the modular nature of HKs, domain shuffling can lead to new HKs and the fusion of the HK and RR genes of an operon can create hybrid HKs [27]. The function of TCS pathways relies on molecular recognition and, consequently, HK and RR sequences have extensively co-evolved [28]. as a means to prevent the disruption of signalling by mutations. Co-evolution also prevents crosstalk with other pathways [21]. Concerning the origin of HKs, it has been suggested that they come from ATPases of the GHKL superfamily: Hsp90, the mismatch repair protein MutL or type II topoisomerases [28]. On the other hand, the origin of the RRs remains unclear beyond a general structural similarity to P-loop NTPases [28]. The evolution of RRs can be the result of changes in the DNA-binding or RNA polymerase interaction sites; it includes lateral gene transfer and gene duplication, while domain shuffling and rearrangement have also led to new forms of RRs [21].

### Evolution of QS

Most of the phylogenetic studies of QS have mainly focused on the produced AIs or on specific pathways, mainly LuxI–LuxR and LuxS–LuxQ. A phylogenetic analysis for the proteins LuxI, LuxR and LuxS, revealed that these proteins are ancient in many bacterial species and that they appeared very early in the evolutionary path of the bacteria [15]. Also, in most cases the gene pairs of the inducer and its cognate response regulator are located next to each other on the chromosomes and they preserve their pairwise function, a sign of common evolutionary history [15]. The existence of homologous proteins in some genomes is representative of horizontal transfer and duplication of the genes, both of which can alter the regulation of different gene targets [15]. In a study of the ComQXPA pathway, the phylogenetic tree of HK proteins in 60 firmicute genomes containing the c*omQXPA* locus clustered all the ComP proteins from various organisms together, instead of gathering them with HKs of the same organism. Therefore, it concluded that ComP evolved from the other HKs before the appearance of the modern bacterial species and thus the ComQXPA pathways have an ancient origin [29].

In a study of the *Vibrionaceae *, it was found that the various inducers (AHLs) do not show any correlation with the species’ geographical distribution. This result indicates that AHLs had a worldwide distribution throughout their evolution, since there was no identification of specific AHL to the environment of the organism which produces it [30]. The results of another survey, focusing on thermophilic bacteria, indicated that some phyla may use AI-2 for QS communication [31]. The phylogenetic tree comparison of LuxS and the 16S rRNA of thermophiles and mesophiles showed that LuxS (which produces the autoinducer) of mesophilic bacteria may originate from thermophiles. Also, the LuxS proteins of thermophilic bacteria within a phylum were evolutionarily closer compared with LuxS of different phyla [31].

To our knowledge, no study has so far addressed the evolutionary relationships of different QS pathways. In this study we examine the evolutionary relationships between different TCS QS systems. We used the KEGG database to identify homologous HKs and their cognate RRs, which are also part of the TCS, and to examine the distribution of each of these QS pathways across the bacterial kingdom. We discuss lineages and species which have more than one QS mechanism and whether all components of each pathway are conserved. In the KEGG database the HKs and their cognate RRs are divided into separate families; we thus also addressed to what extent each of the QS HK and RR protein families can be distinguished on the basis of the presence of distinct functional domains. Finally, we have used phylogenetics of the HKs and RRs of different types and families to examine whether some QS pathways are more closely related to others.

## Methods

**Fig. 1.**
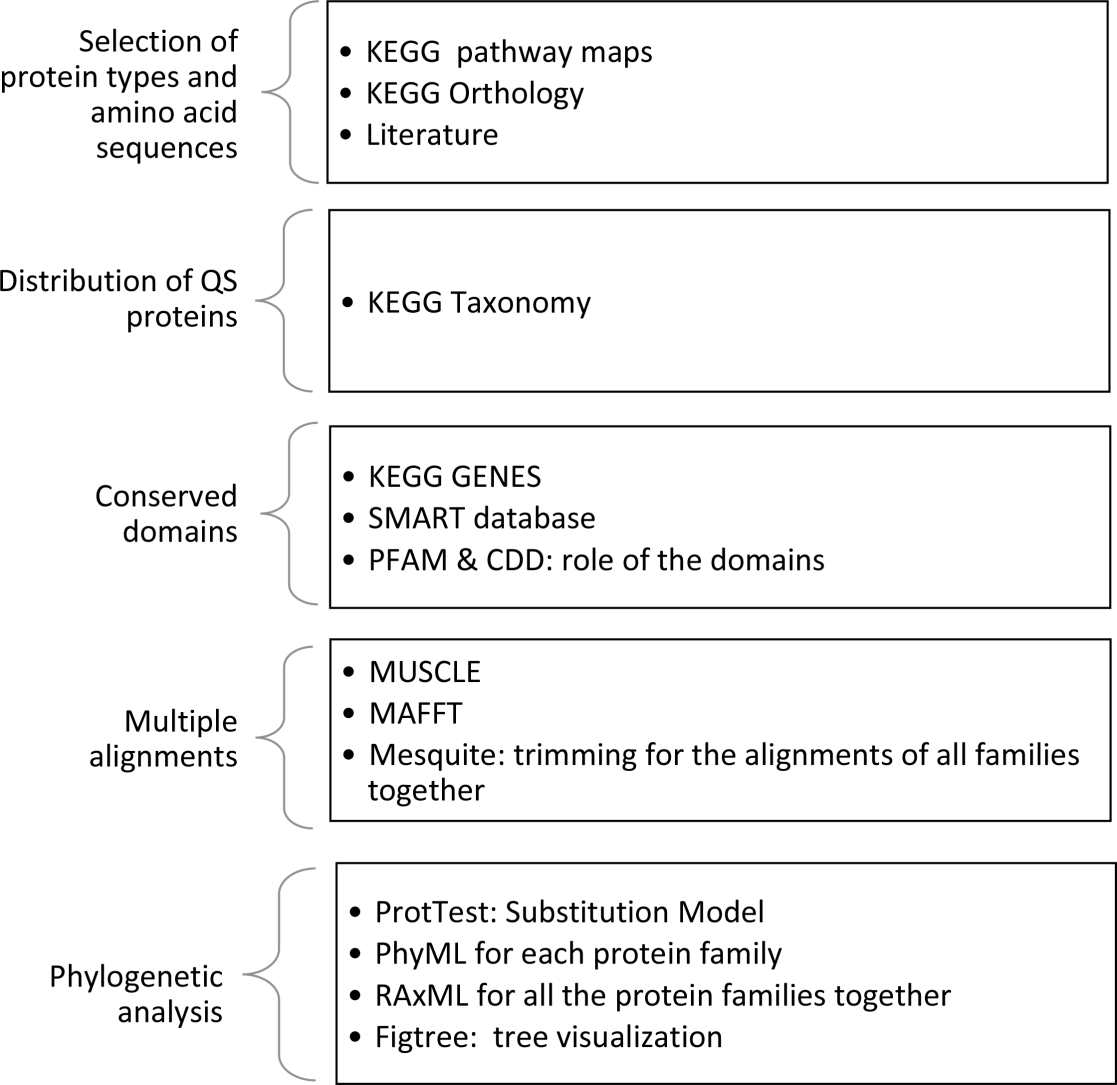
An overview of the methods used in this paper.

An overview of the implemented methods is presented in Fig. 1.

### Distribution of QS proteins

We used the KEGG Database (Kyoto Encyclopedia of Genes and Genomes, https://www.kegg.jp/ or https://www.genome.jp/kegg/), to select the pathways and amino acid sequences used in this study. First, the two component system protein types that are also part of the quorum sensing system were selected using the pathway maps of the KEGG PATHWAY database (maps ko02020 and ko02024 respectively, Figs S1 and S2, available in the online version of this article). We then checked for the presence of the protein subunits of the selected TCS QS pathways in all the major bacterial lineages (based on [32]), which represent various phyla, classes and genera, as shown in Table S1. To do this, we used the orthology group accession of each protein type from the KEGG ORTHOLOGY database to analyse the lineages in which each protein type is present, by selecting ‘Taxonomy’ within the ‘Genes’ section of each orthology page (e.g. https://www.genome.jp/entry/K07706).

### Conserved domains

One amino acid sequence for each protein type was chosen randomly to identify the conserved domains on it, by searching in the ‘Motifs’ section of the KEGG GENES database. We also used Simple Modular Architecture Research Tool (SMART, http://smart.embl-heidelberg.de/) to identify additional domains not shown in KEGG. In general, most of the domains were the same in the two databases. Both SMART and KEGG give the E-value of each conserved domain; the domains that were finally taken into consideration for our research, were those with an E-value of at least 10^−5^ which were also mentioned as ‘Confidently predicted domains’ in SMART. This E-value cut-off was chosen on the basis of previous publications [33–35] and as suggested by the NCBI Conserved Domain Database (CDD) Help page (https://www.ncbi.nlm.nih.gov/Structure/cdd/cdd_help.shtml#GlobalOptions). Next, using the Pfam database database (https://pfam.xfam.org/), we found the clans in which these domains are classified (Table S2) as well as information about their function. For further information we used the CDD of the NCBI (https://www.ncbi.nlm.nih.gov/Structure/cdd/cdd.shtml).

### Phylogenetic analysis

For the phylogenetic analysis, HK and RR amino acid sequences of the selected TCS QS pathways were retrieved in FASTA format from the KEGG database. The number of sequences for each protein type was kept as low as possible so as to have a manageable amount of data but at the same time include a representative portion of the different taxa and families. The average number of sequences used for phylogenetic analysis for each protein type was thus about 20. The actual number of sequences analysed, and the percentage representation of taxa, families and species out of the total available ones in the KEGG database are given in Tables S3a, b. Accession numbers and abbreviations for all sequences used are in the dataset.

Multiple sequence alignments were created using the MAFFT (Multiple Alignment using Fast Fourier Transform, https://www.ebi.ac.uk/Tools/msa/mafft/) [36] and muscle (MUltiple Sequence Comparison by Log- Expectation, https://www.ebi.ac.uk/Tools/msa/muscle/) [37] alignment programmes, with default settings, for (a) each HK family, (b) all the HKs, (c) each RR family and (d) all the RRs

The substitution model for each alignment was inferred via the ProtTest3.4 programme (https://github.com/ddarriba/prottest3/releases) [38]. In order to compute the likelihood scores, the following settings were used: Substitution matrices to test: VT, Blosum62, JTT, Dayhoff, WAG; Rate variation: I, G, I+G; Amino acid frequencies: unchecked empirical. The results were evaluated by the Akaike information criterion (AIC). More specifically, the substitution model for both the muscle and MAFFT multiple alignments of the HKs and RRs was VT.

Phylogenetic reconstruction for each protein family was done with PhyML at http://www.phylogeny.fr/ (also available at https://ngphylogeny.fr/) [39]. The ‘A la Carte’ function was used with the following settings: ‘remove gaps in alignment’ (alignment curation), maximum likelihood method (PhyML), SH-like approximate likelihood-ratio test (aLRT) as the statistical test for branch support and 100 bootstraps. For each multiple alignment we chose the WAG substitution model as the best one according to the ProtTest results (WAG, JTT and Dayhoff were the given options in the PhyML settings).

The phylogenetic trees for all the protein families together (two trees: HKs and RRs) were reconstructed in the CIPRES Science Gateway (https://www.phylo.org), using RAxML (Randomized Axelerated Maximum Likelihood): *RAxML-HPC BlackBox (8.2.12) - Phylogenetic tree inference using maximum likelihood/rapid bootstrapping on XSEDE* [40], using the VT substitution model and 100 bootstraps. Before entering the multiple alignments into the programme, we removed any positions not conserved in more than 50 % of the sequences. For this purpose, we used Jalview (https://www.jalview.org/) to detect those positions and then the Mesquite programme to do the trimming (http://www.mesquiteproject.org/).

The multiple alignment for all the HK proteins consisted of 244 sequences and had a total length of 2106 amino acids using MAFFT or 1806 using muscle. In both alignments, there were several clusters of conserved residues towards the N-terminal region of the alignment (MAFFT: 64–808, muscle: 90–528) and near the C-terminal part (MAFFT: 1073–1529, muscle: 888–1307). In the MAFFT alignment, the clusters of the conserved residues were more dispersed than in the muscle alignment. The trimmed MAFFT alignment had 378 amino acids (Fig. S3), while the trimmed muscle alignment had 389 amino acids (Fig. S4).

The multiple alignment for all the RR proteins consisted of 196 sequences and had a total length of 713 amino acids using MAFFT or 670 using muscle. Both the muscle and MAFFT alignments contained four clusters of conserved residues (MAFFT: 101–245, 443–481, 525–546, 639–672, muscle: 63–215, 368–432, 486–504, 598–637). Trimming resulted in an alignment of 217 amino acids using MAFFT (Fig. S5), and 216 using muscle (Fig. S6).

All phylogenetic trees were visualised with the Figtree programme (https://github.com/rambaut/figtree/releases). The results were saved as PNG images and were further edited with Microsoft Draw. Nodes were labelled as white, grey or black bullets, if their bootstrap value was 50–79 %, 80–94% and 95–100% respectively. The raw and masked alignments, as well as the phylogenetic trees in nexus format are available in the Dataset. The alignments for each family and all families together are available in the files ‘HK alignments’ and ‘RR alignments’. The alignments of all HKs and all RRs are presented as they were masked in Mesquite: in the added taxon, ‘C’ and ‘I’ represent the removed and included positions, respectively. The trees in nexus format are available in the ‘HKs trees’ and ‘RRs trees’ files.

## Results

### Distributions of QS proteins

**Fig. 2.**
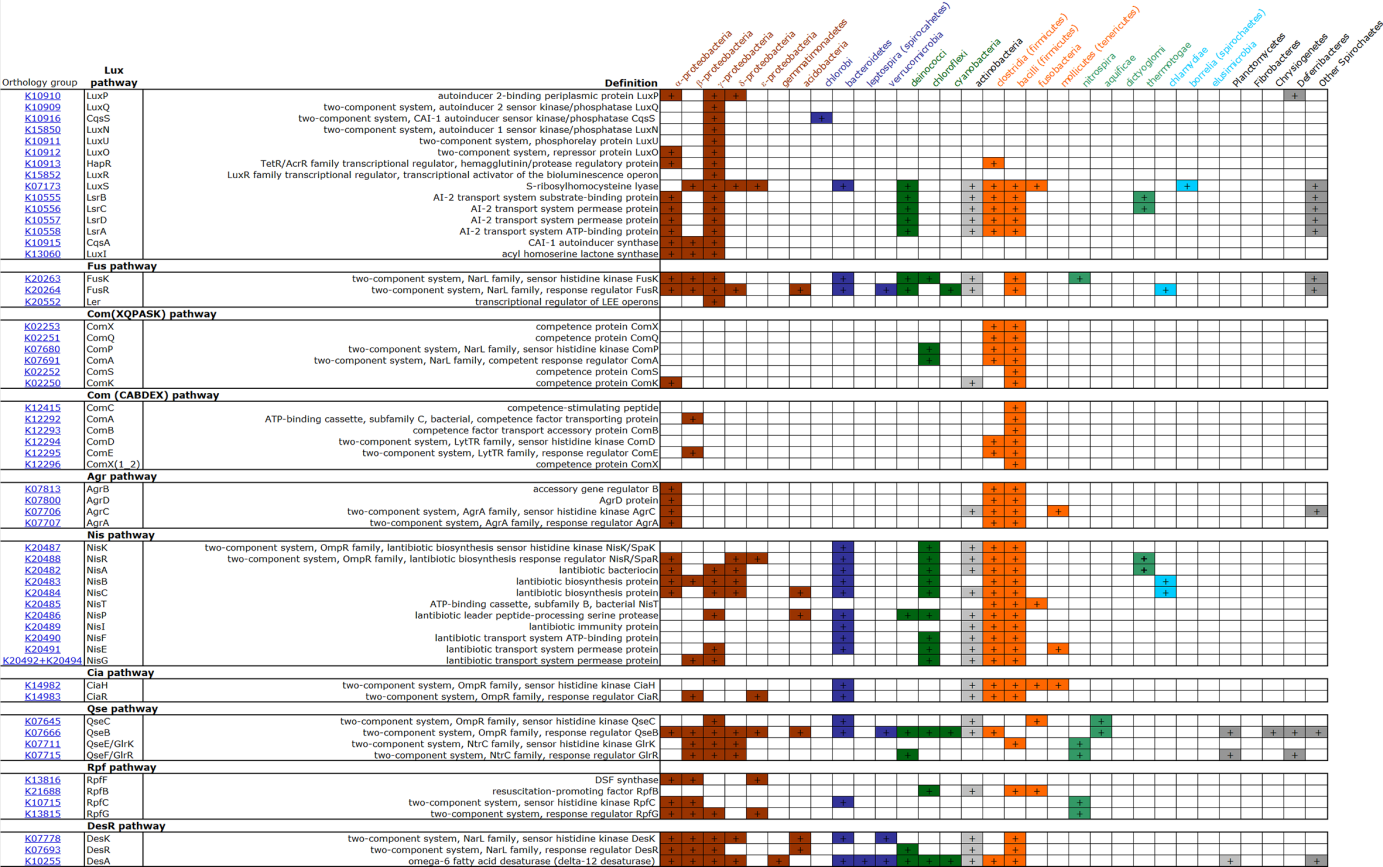
Presence of the protein subunits of different QS pathways in selected groups of bacteria. Coloured sectors indicate presence, empty sectors indicate absence, based on the KEGG taxonomy database for each orthology group. ‘Other’ Spirochaetes refer to he genera *Treponema *, *Spirochaeta *, *Leptospira* and *Salinispira *.

**Fig. 3.**
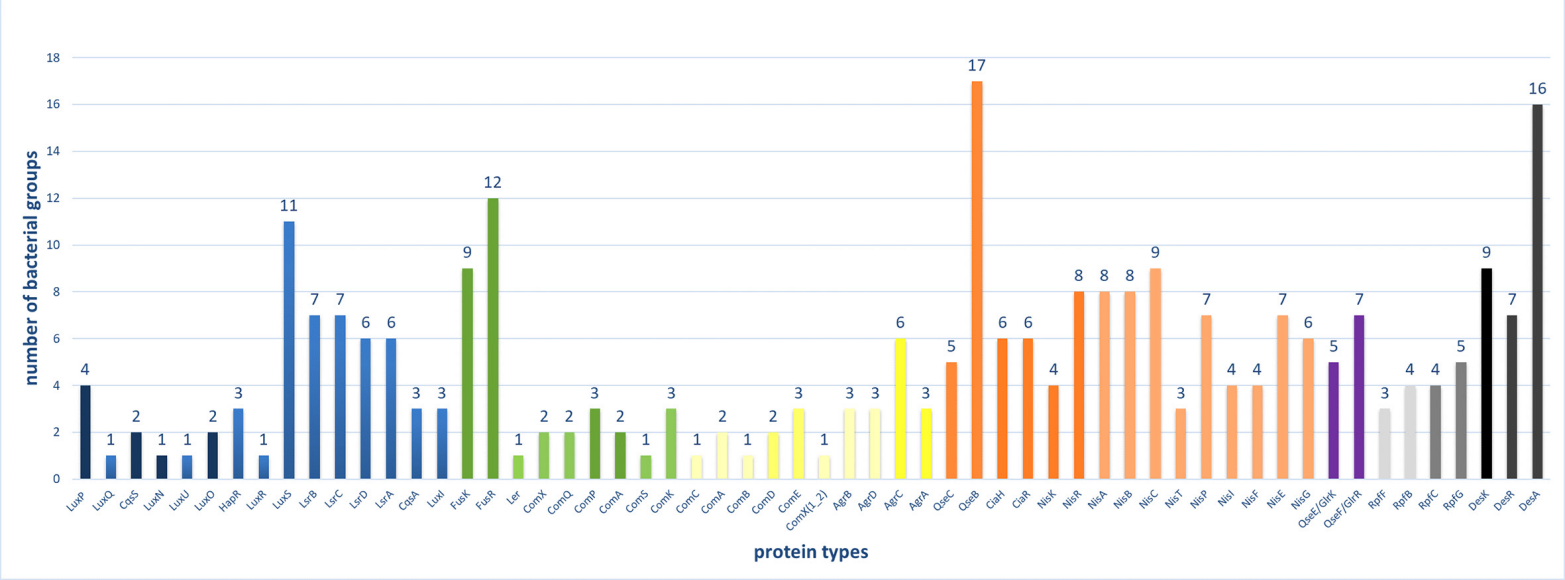
The number of bacterial groups in which each protein is found. The different colours indicate the family of each HK and RR in our study; proteins involved in the same pathway are indicated with a lighter shade of the same colour. Blue: Lux family, Green: NarL family, Yellow: LytTR family, Orange: OmpR family, Purple: NtrC family, Grey: ‘Other’, Black: Des pathway.

We used the KEGG PATHWAY database to select the pathways for our analysis based on the criterion that they belong both to two component systems and quorum sensing systems (Figs S1 and S2). A brief description of the regulated functions of each HK–RR pair of the selected TCS QS pathways is presented in Table 1. The distribution of the proteins comprising the selected TCS QS pathways across bacterial lineages is demonstrated in Fig. 2; bacterial groups are shown in different colours based on those used previously [32]. The majority of the complete pathways are found in γ-proteobacteria, clostridia and bacilli. A significant number of proteins are also found in α-, β-, δ- and ε-proteobacteria, bacteroidetes and actinobacteria. Also, based on the number of bacterial groups which contain proteins of each pathway, the most widespread proteins are those of the DesR, Fus, Nis pathways and the Lsr proteins of the Lux pathway. A more limited distribution across taxa is seen for the proteins of the two Com pathways and the Agr pathway. Looking at each protein separately the most widespread proteins are QseB, DesA, FusR and LuxS (Fig. 3). Interestingly, those belong to different pathways (QseB: Qse, DesA: DesR, FusR: Fus and LuxS: Lux pathways). The Lux pathway contains the most widespread as well as some of the most limited pathway components (LuxQ). There is also a difference in the distribution of the HKs and their cognate RRs: In most cases, the RRs are found in more bacterial groups than their cognate HKs (FusR vs FusK, ComE vs ComD, QseB vs QseC, NisR vs NisK, GlrR vs GlrK, and RpfG vs RpfC).

**Table 1.** A brief description of the functions regulated by each HK and RR pair included in our study

Pathway-regulated functions		
**LuxQ–LuxU–LuxO**	**LuxN–LuxU–LuxO**	**CqsS–LuxU–LuxO**
Detection of AI-2, HAI-1 and CAI-1 by LuxQ, LuxN and CqsS respectively Bioluminescence, biofilm formation and virulence in *Vibrio* species [57].		
**ComP–ComA**		
ComP senses ComX pheromone (10-amino-acid modified peptide) in *Bacillus subtilis* [58] Induces competence, extracellular DNA release, biofilm formation, synthesis of a lipopeptide antibiotic surfactin (biosurfactant and antiviral) [12] Controls a series of genes in *B. subtilis,* either directly through ComA or indirectly, that are responsible for various biological functions [58].		
**FusK–FusR**		
FusK senses fucose Control of virulence and metabolic genes of enterohemorrhagic *E. coli* [11].		
**QseC–QseB**		
Sensing of norepinephrine, epinephrine and AI-3 [9] Virulence, flagella activation, assembly and motility, regulation of the TCS QseE-QseF [59].		
**CiaH–CiaR**		
Sucrose-dependent biofilm formation, competence and stress tolerance in *Streptococcus mutans* [56]. Competence, cefotaxime susceptibility, autolysis, bacteriocin production, oxidative stress resistance and virulence in *Streptococcus pneumoniae* [60].		
**NisK–NisR**		
Detection of extracellular nisin Self-regulation of nisin production [61], a lantibiotic type produced by lactic acid bacteria [62] and virulence in *Streptococcus suis* serotype 2 [63].		
**AgrC–AgrA**		
AIP acts as autoinducer [64] Virulence [65], biofilm formation in *Listeria monocytogenes* [66], positive feedback loop by the activation of the transcription of the agr QS genes, expression of virulence genes in *Staphylococcus aureus* [67].		
**ComD–ComE**		
ComD binds to competence stimulating peptide (CSP) Initiation of competence, positive feedback loop of the QS components in *S. pneumoniae* [68], biofilm formation in *S. mutans* [53].		
**GlrK–GlrR/QseE–QseF**		
QseE binds to host epinephrine and norepinephrine Controls virulence and cell envelope functions in Enterobacteraceae. In a study on *E.coli* strain K12, protein QseG is essential for the activation of the HK QseE [69].		
**RpfC–RpfG**		
Activation of RpfC by diffusible signal factor (DSF) signals including DSF, BDSF, CDSF and IDSF [70] Virulence for *Xanthomonas oryzae* [71] and *Xanthomonas campestris pv. campestris* [72].		

All pathways are fully present in at least one bacterial group, but many lineages have only some of the subunits of each pathway. The Lux system contains a pathway of HKs and an RR, and the cytoplasmic Lsr proteins; both pathways are activated by the AI produced by LuxS. All Lux pathways are fully present in γ-proteobacteria. deinococci, actinobacteria, clostridia, bacilli, α- and γ-proteobacteria, and spirochaetes possess most or all proteins of the Lsr protein pathway. All three proteins of the Fus pathway are present in γ-proteobacteria; however, the HK–RR pair is found also in α- and β-proteobacteria, bacteroidetes, deinococci, actinobacteria and spirochaetes. All proteins of the Com(XQPASK) pathway are found in bacilli and most proteins are found in clostridia. Also, the HK–RR pair ComP–ComA is present in deinococci. The proteins of Com(ABDEX) are all found in bacilli, whereas the HK–RR pair ComD–ComE is present in clostridia. The Agr pathway (both AgrC and AgrA) is fully present in α-proteobacteria, clostridia and bacilli. All the proteins of the Nis pathway are present in both clostridia and bacilli, while bacteroidetes, chloroflexi and actinobacteria contain all but two of the proteins. Other bacterial groups contain parts of the pathway but not the full sensing system (they lack either one or both the HK and RR). Bacteroidetes, actinobacteria, clostridia and bacilli contain both the CiaH and CiaR proteins. Concerning the Qse pathway, γ-proteobacteria contain all of the proteins [QseC–QseB and QseE(GlrK)–QseF(GlrR)]. QseC–QseB are present in bacteroidetes, actinobacteria and aquificae. QseE(GlrK)–QseF(GlrR) are found in β- and δ-proteobacteria and *Nitrospira *. No bacterial group contains all the proteins of the Rpf pathway but α- and β-proteobacteria and *Nitrospira* contain the HK–RR pair RpfC–RpfG. All three proteins of the DesR pathway are present in α-, β- and γ-proteobacteria, actinobacteria and bacilli. For further details of all other bacterial groups which contain only certain proteins of each pathway, please refer to Fig. 2.

### Conserved domains

In the Pfam database, domains are organised into larger groups called ‘clans’; the NCBI database characterises most domains as ‘superfamilies’. Table 2 shows the clans and their corresponding domains, which are found in the studied sequences, and Table S2 shows the accessions for each domain in PFAM and InterPro, as well as the entry of the superfamily (NCBI-CDD) and pfam clan they belong to. The following domains do not belong to any clan: MASE1, PilJ, Hpt, Sigma54_AID, Trans_reg_C. An overview of the conserved domains of both HKs and RRs is present in Table 3.

**Table 2.** Categorization of the conserved domains of all the HK and RR types in this study

Clans	Domains				
**HTH (Helix–Turn–Helix**)	HTH_8	Sigma70_r4_2	GerE	Sigma54_CBD	Sigma54_DBD
**His kinase A**	HATPase_c	HATPase_c_5	HisKA	HisKA_3	
**P-loop-NTPase**	AAA	AAA_5	Sigma54_activitat	Sigma54_activ_2	
**Cache-like domains**	2CSK_N				
**CheY-like superfamily**	Response_reg Receiver domain of RRs				
**HD-PDEase**	HD	HD_5			
**Clp crotonase**	ECH_1 Enoyl-CoA hydratase	ECH_2 Enoyl-CoA hydratase			
**Periplas BP**	Periplas_BP				
**Tudor**	LytTR DNA-binding domain				
**YbjQ-like**	RcsF lipoprotein				

**Table 3.** Occurrence of the conserved domains of all the HKs and RRs in the different protein types and families

Domain	Protein types	Family
**HATPase_c**	11	All of the families
**HATPase_c_5**	3	LytTR
**HisKA**	6	OmpR, NarL, Lux, Other, NtrC
**HisKA_3**	1	NarL
**Response_reg**	15	All of the families
**Hpt**	2	Lux, Other
**Trans_reg_C**	3	OmpR
**LytTR**	3	LytTR
**GerE**	2	NarL
**Sigma70_r4_2**	1	NarL
**Sigma54_activat**	2	LuxO, NtrC
**Sigma54_activ_2**	2	LuxO, NtrC

#### Conserved domains in HKs

**Fig. 4.**
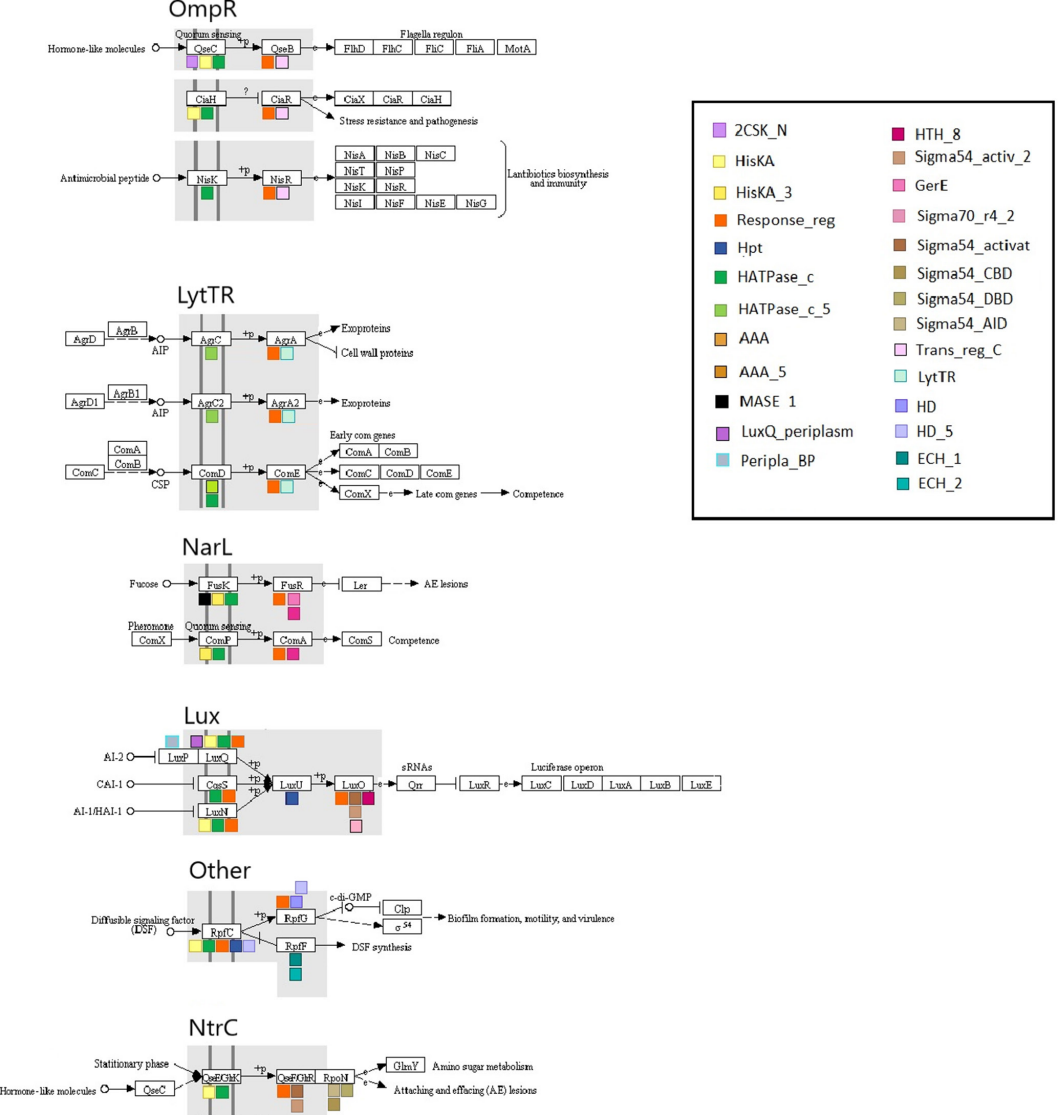
An overview of the position of the conserved domains in the HKs and RRs of this study. The similar colours of domains indicate their presence in a common clan of the Pfam database. The pathway drawings are from KEGG PATHWAY map ko02020, edited with Microsoft Draw.

Based on the sequences analysed, the length of the HKs varies from 131 (ers1AgrC) to 960 (pprLuxN) amino acids. The longest HKs are the hybrid ones (Lux family: LuxN, LuxQ and CqsS, ‘Other’: RpfC) which also contain more conserved domains. Differences in the length of the sequences can also be found within the proteins of the same family. The HK LuxQ functions as a complex with LuxP. Most HKs contain one to three conserved domains and up to six (Fig. 4). The members of the OmpR, LytTR and NtrC (GlrK) families have the fewest conserved domains (one or two), whereas the HKs of the Lux and ‘Other’ (RpfC) families have more than two conserved domains (Fig. 4).

The common trait is the existence of either HATPase_c or HATPase_c_5, which are also the only conserved domains in the proteins of the LytTR family and in NisK of the OmpR family. According to NCBI-CDD, Histidine kinase-like ATPase domains (HATPase domains), also referred to as GHKL ATPase domains, are found in ATP-binding proteins, including histidine kinases. Concerning the N-terminal region, only three proteins (QseC, LuxQ and FusK) contain conserved domains, which belong to the OmpR, Lux and NarL families, respectively. These are Cache-like sensory domains (2CSK_Nand LuxQ-periplasm), and MASE1 (PFAM: predicted integral membrane sensory domain in HKs, diguanylate cyclases and other bacterial signalling proteins). The response regulator receiver domain or phosphoacceptor receiver-REC (Response_reg) and Hpt domains are located in the C-terminal region of hybrid HKs. The Response_reg receives the signal from the HK and histidine-containing phosphotransfer domain (Hpt), which mediates phosphotransfer reactions in multi-step TCS signalling systems.

#### Conserved domains in RRs

The average length of RRs is significantly shorter than that of the HKs. For the sequences analysed here, it varies from 80 (srqComE) to 508 (cijLuxO) amino acids. Most RRs contain two conserved domains except for LuxO, which has three domains (Fig. 4). The N-terminal regions always contain the response_reg domain, which is a common structure trait for all the RRs. The conserved effector domain in the C-terminal region is different in each RR family. The only exception is the protein GlrR (NtrC family), which has the same conserved domain as LuxO (Lux family). The majority of these effector domains have a DNA-binding activity: HTH_8, Sigma70_r4_2, GerE, and Sigma54_DNA-binding domain (DBD) are helix–turn–helix (HTH) domains. Although Sigma54_core binding domain (CBD) is also considered to be a HTH domain, it interacts directly with the core RNA polymerase, forming an enhancer-dependent holoenzyme. The centre of this domain is slightly similar to a HTH motif, which may represent a DNA-binding domain. Other effector domains are P-loop-NTPases: Sigma54_activat, Sigma54_activ_2 interact with the sigma-54 factor of RNA polymerase; AAA and AAA_5 (ATPases associated with a variety of cellular activities) are described as a dynein-related subfamily and although their role is yet unclear, the AAA+superfamily to which AAA ATPases belong, is associated with chaperone-like functions. Sigma_54_activator interacting domain (Sigma_54_AID) is not classified into any clan. It is necessary for the interaction of Sigma54 RNAP holoenzyme with the activator and it can also inhibit transcription initiation prior to interaction with the activator.

### Phylogenetic trees by family

Given the families formed by the HKs and their cognate RRs, we reconstructed four phylogenetic trees for the HKs and three for the RRs. The HKs GlrK (NtrC family), RpfC (‘Other’), their cognate RRs GlrR and RpfG, as well as LuxO are the only members of their family, hence no phylogenetic tree was inferred. Unless otherwise stated, only well-supported clades, with >90 % bootstrap support, are discussed below. Phylogenetic reconstructions were done with PhyML using MAFFT alignments, but the major differences between the trees created from the MAFFT and muscle alignments (Figs S7–S12) are also mentioned, wherever they apply.

#### The NarL family

**Fig. 5.**
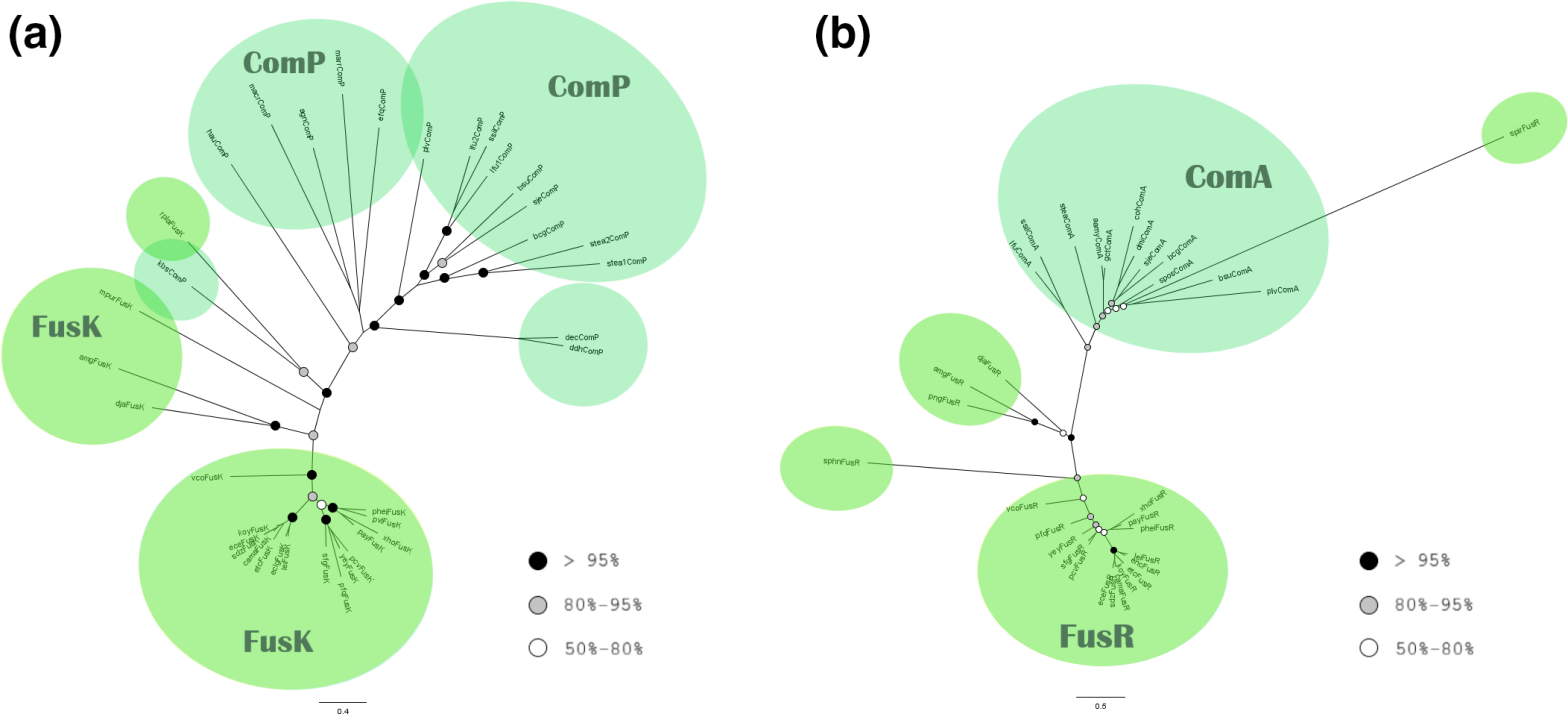
Phylogenetic analysis of the NarL family. (**a**) Histidine kinases. (**b**) Response regulators. Both trees were reconstructed using PhyML based on the MAFFT alignment. The colours of the circles indicate the bootstrap value of the nodes. White: 50–79 %, Grey: 80–94 %, Black: over 95 %.

The majority of the FusK and ComP sequences are organised in distinct clades in the PhyML tree. Each clade consists of sequences of the same protein type, supported by high bootstrap values (Fig. 5a). The same structure is observed in the phylogenetic tree of their cognate RRs (Fig. 5b), also supported by good bootstrap values, although most of them are below 95 %. Notably, the FusK and FusR amino acid sequences are both organized in less dispersed clades than ComP and ComA. The overall structure of the corresponding muscle trees is almost identical for both HKs and RRs (Fig. S7a, b) but with lower bootstrap values at the centre of the tree.

#### The OmpR family

**Fig. 6.**
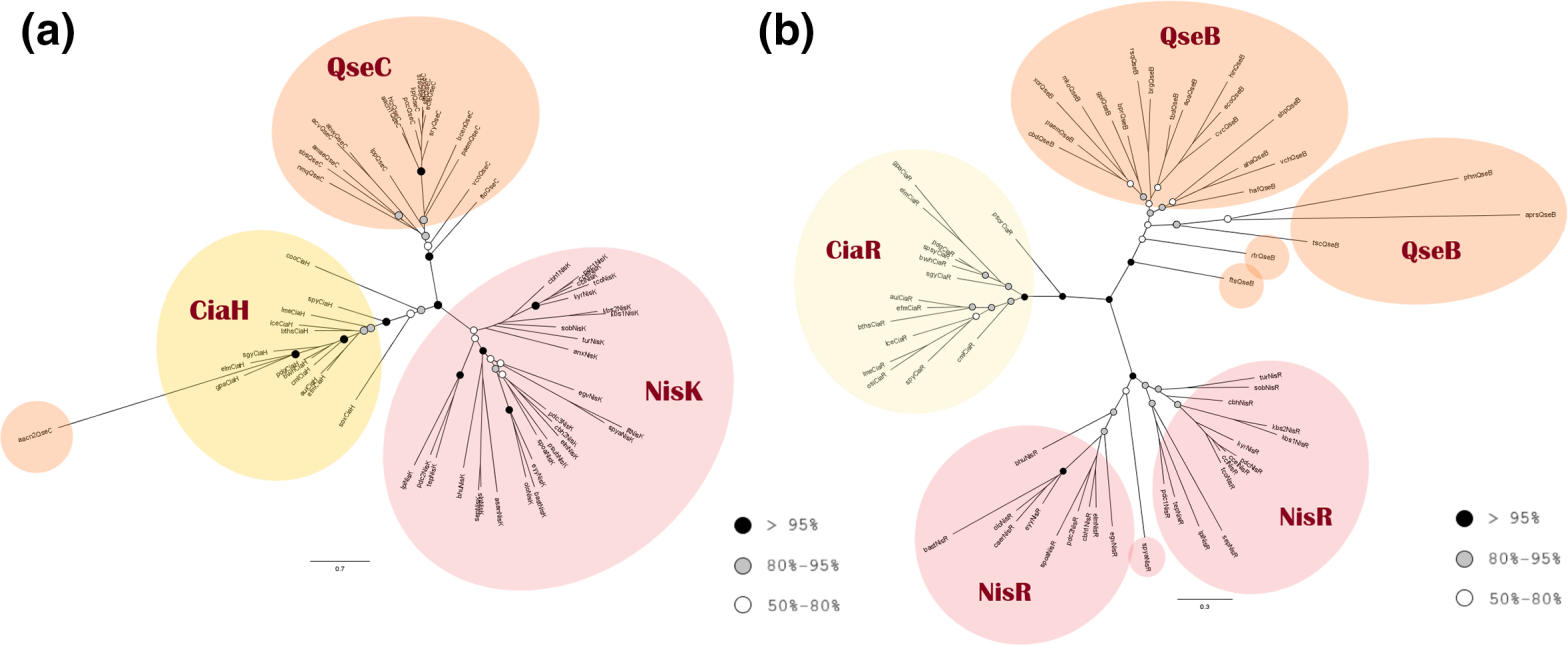
Phylogenetic analysis of the OmpR family. (**a**) Histidine kinases. (**b**) Response regulators. Both trees were reconstructed using PhyML based on the MAFFT alignment. The colours of the circles indicate the bootstrap value of the nodes, as in Fig. 5.

All different HK types of the OmpR family group into separate clades. None of the HK sequences is misplaced in a clade of a different protein type, except aacn2QseC, which falls within the CiaH clade. Their cognate RRs are also separated according to protein type. There is no group formed between two of the three protein types, as no clade is closer to one of the other two protein types in both HKs and RRs (Fig. 6a, b). While in the NisK clade the taxa are close together, the clade of the NisR RRs consists of two large distinct clades. A very similar structure is observed in the trees based on the muscle alignment (Figs S8a, b).

#### The Lux family HKs

**Fig. 7.**
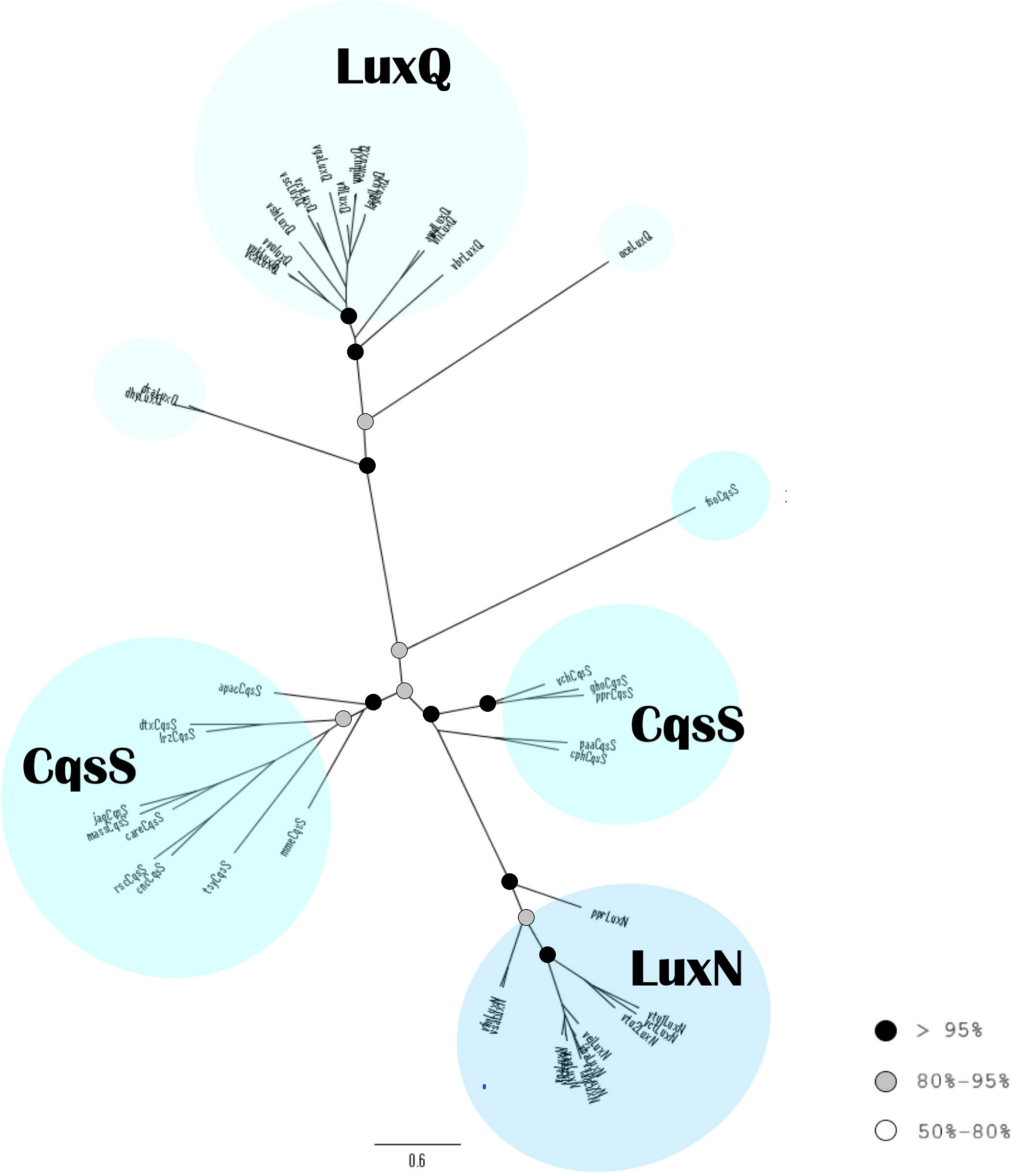
Phylogenetic analysis of the Lux family histidine kinases. Tree reconstructed using PhyML based on the MAFFT alignment. The colours of the circles indicate the bootstrap values of the nodes, as in Fig. 5.

The sequences of each HK type of the Lux family (LuxN, LuxQ and CqsS) are grouped into distinct clades (Fig. 7). The LuxN sequences have one common node. On the other side of the tree, most LuxQ sequences create a common clade. The CqsS sequences branch out at the centre of the tree into three clades. In contrast, most LuxQ branches in the PhyML tree based on the muscle alignment (Fig. S9) appear near the core of the tree; it seems that the clades CqsS and LuxQ switch positions in the MAFFT and muscle versions of the tree. As there is only one RR type for all the Lux family HKs (LuxO), there is no phylogenetic tree for the RRs of the Lux family.

#### The LytTR family

**Fig. 8.**
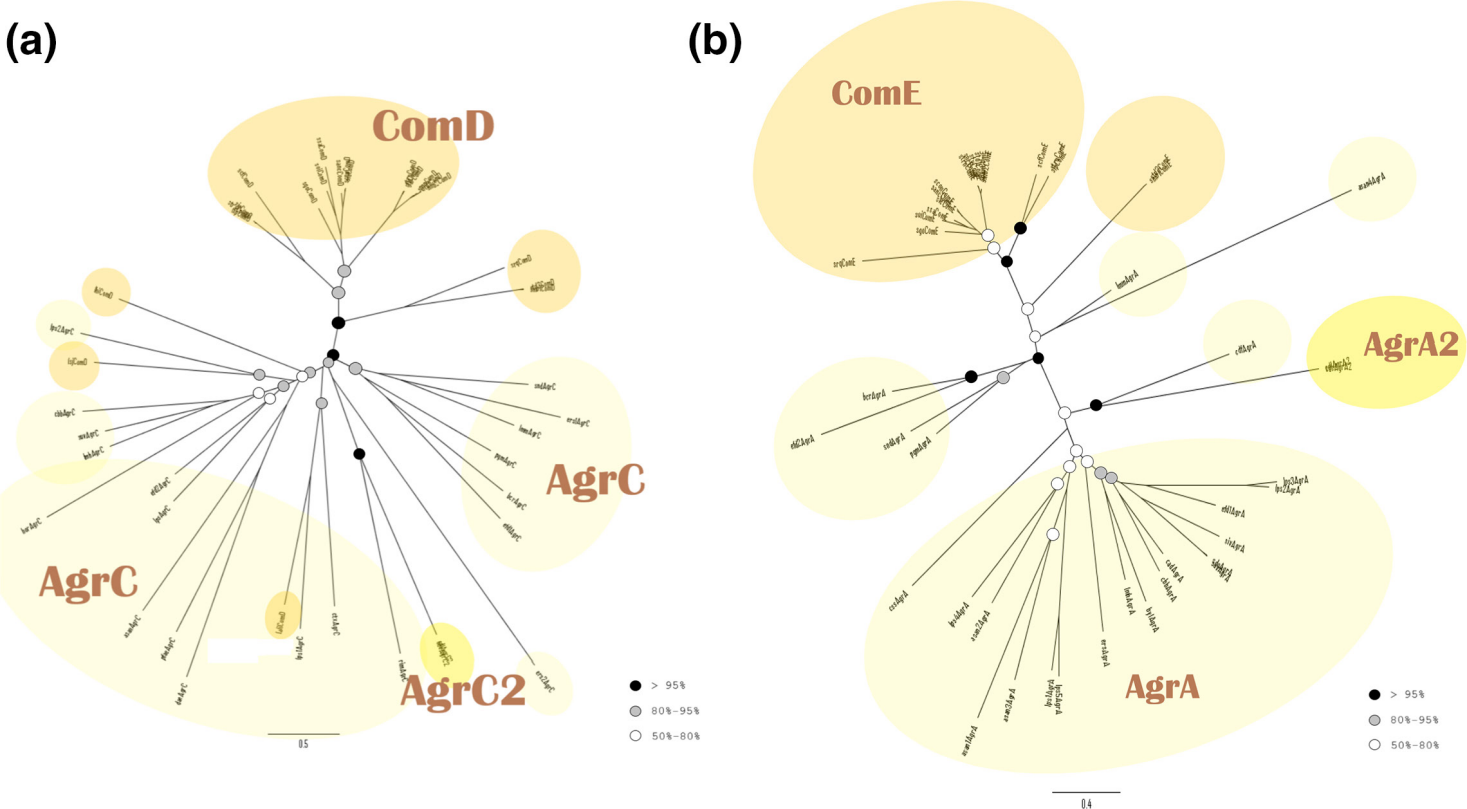
Phylogenetic analysis of the LytTR family. (**a**) Histidine kinases. (**b**) Response regulators. Both trees were reconstructed using PhyML based on the MAFFT alignment. The colours of the circles indicate the bootstrap values of the nodes, as in Fig. 5.

The different types of HKs of the LytTR family, as well as their cognate RRs, are clearly separated from each other and both phylogenetic trees have a similar overall appearance (Fig. 8a, b). The AgrC sequences form several clades, all of which stem from a common node with high bootstrap support. The two AgrC2 sequences are found in a distinct clade within the AgrC clade. Similarly the AgrA RR sequences form multiple clades, and the AgrA2 sequences are found in a distinct clade near the AgrA clades. Most of the ComD sequences are gathered in one clade, apart from some sequences placed among AgrC sequences. The ComE RRs create a main common clade, with the exception of a smaller clade, which branches earlier.

### Phylogenetic trees of all the families together

#### Phylogenetic analysis of all the HK families

**Fig. 9.**
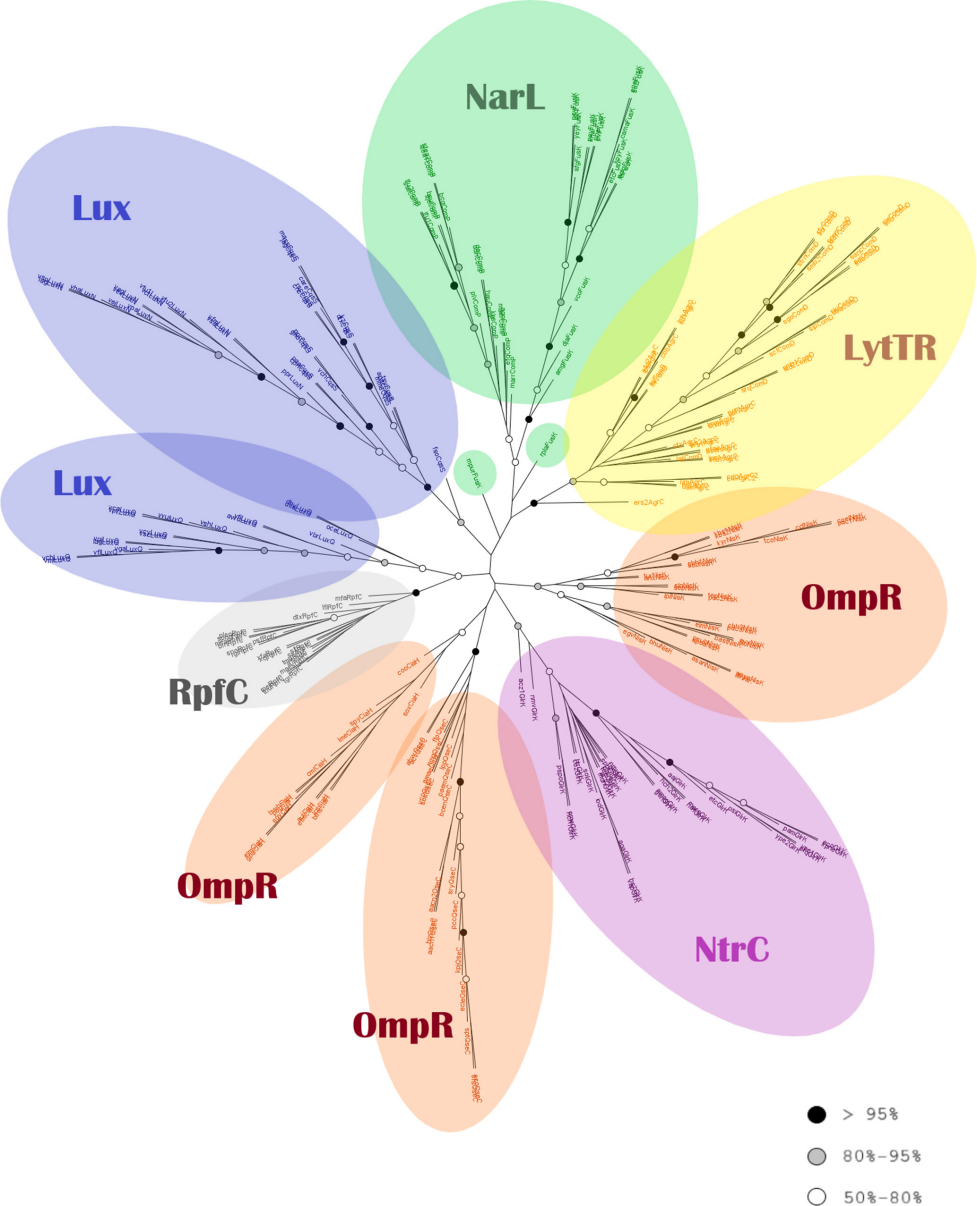
Phylogenetic analysis of all histidine kinase families. Tree reconstructed using RaxML based on the MAFFT alignment. The colours of the circles indicate the bootstrap values of the nodes, as in Fig. 5.

The two phylogenetic trees of all the HKs and RRs families together are discussed below, based on the MAFFT alignment. Major differences from the phylogenetic trees based on the muscle alignments are also mentioned; overall, the RAxML trees based on the MAFFT alignment yielded better bootstrap values. In general, the members of each HK family tend to form one main clade, although the bootstrap values were not always significant. The clades of each HK protein type are also mostly monophyletic (Fig. 9). The sequences of the NarL and LytTR families, as well as the RpfC (‘Other’) and GlrK HK types (NtrC family) are each gathered in one clade, whereas the protein sequences of the Lux and the OmpR family are found in more than one clade. The following three groups of families are discernible: (1) LytTR and NarL, (2) OmpR and NtrC, (3) Lux (LuxQ) and ‘Other’ (RpfC).

##### NarL family

The sequences of this family form two clades, distinguishing the FusK (99 % bootstrap) and ComP (50 % bootstrap) sequences, which meet at a common node (78 % bootstrap). Only rplaFusK and mpurFusK branch earlier (Fig. 9). A similar pattern of the NarL clade in seen in the tree based on the muscle alignment, but with lower bootstrap values (Fig. S11).

##### OmpR family

The HK types of this family are separated into three main clades, one each for QseC, CiaH and NisK sequences (Fig. 9). The clades of QseC and CiaH share a common node (41 % bootstrap), while NisK is separate and closer to GlrK (NtrC family). Interestingly, the tree resulting from the muscle alignment presents CiaH together with NisK (40 % bootstrap), while QseB is separate and closer to GlrK, but with a very low bootstrap value (Fig. S11).

##### Lux family

Two distinct clades are formed by the HKs of the Lux family: one clade, near the NarL–LytTR group, consists of CqsS and LuxN sequences, which are mostly separated from each other into distinct clades. The other is made of only LuxQ sequences and groups with the RpfC clade (Fig. 9). Although this pattern is also seen in the tree based on muscle, there are sequences separate from the family (Fig. S11). The clades of the Lux family follow the groupings of the phylogenetic tree of the Lux family alone (Fig. 7), as CqsS and LuxN are grouped closer together, while the LuxQ sequences are separate.

##### LytTR family

All the sequences of this family derive from a common node (51 % bootstrap support) (Fig. 9). Most ComD sequences form one clade (78%) within the AgrC clade. The AgrC sequences form many small clades, including the two AgrC2 sequences. The closest clade to the LytTR family is the clade of the NarL family, deriving from the same node with 41 % bootstrap support.

##### NtrC family (GlrK/QseE) and ‘other’ (RpfC):

All the sequences of the GlrK (QseE) HK are in one clade, with 91 % bootstrap support and all RpfC sequences are placed in one clade, with 97 % bootstrap value, neighbouring the LuxQ clade (Fig. 9).

### Phylogenetic analysis of all the RR families

**Fig. 10.**
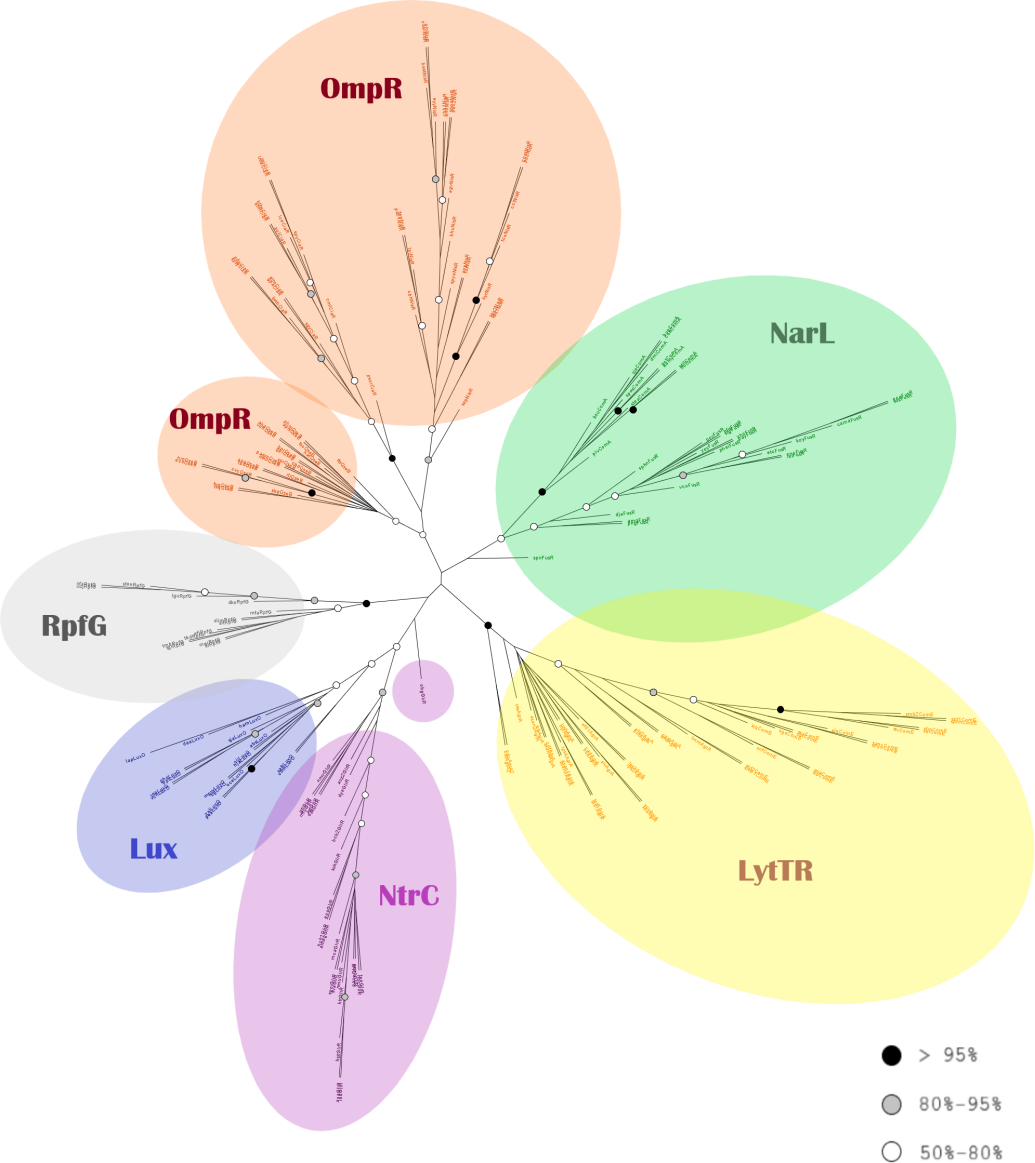
Phylogenetic analysis of all response regulator families. Tree reconstructed using RaxML based on the MAFFT alignment. The colours of the circles indicate the bootstrap values of the nodes, as in Fig. 5.

The RR families are distinctly separated from each other in the phylogenetic tree. Also, the sequences of each RR type within each family remain together, like in the separate phylogenetic trees of each family. No sequence falls outside of the clade of their family or RR type, except obgGlrR (Fig. 10). An overview of the tree reveals a closer relationship between the following family groups: (1) the RpfG, Lux and NtrC families, and (2) the NarL, LytTR and OmpR families.

#### NarL family

Similar to the HKs, the NarL clade consists of two separate clades for each protein type. It shares a common node with the OmpR family but with a very low bootstrap value.

#### OmpR family

The members of the OmpR family are grouped together (68 % bootstrap value) and there is one individual clade for each protein type, supported by significant bootstrap values (except QseB: 52 %). This structure recapitulates the pattern seen in Fig. 6(b). CiaR and NisR group closer together but with just 39 % bootstrap value.

#### Lux family

The Lux family (LuxO) groups together with 65 % bootstrap support, close to NtrC, forming a common clade (52 % bootstrap). The Lux–NtrC clade shares a common node with the RpfG clade (41 % bootstrap).

#### LytTR family

The clade of all the RRs of the LytTR family is well supported, and lies between the NarL and RpfG clades. It consists of many smaller clades or individual branches. The AgrA2 sequences branch out earliest of all and they have a common node with the AgrA clade (100 % bootstrap), whereas the ComE is the last clade. In the tree based on the muscle alignment, the LytTR family clade shares a common node with RpfG (Fig. S12).

#### NtrC family (GlrR protein) and ‘other’ (RpfG protein)

The GlrR (NtrC) sequences group together with a bootstrap value of 83 % and share a common node with the Lux family clade. The RpfG RRs form one clade with 99 % bootstrap value, and share a common node with the Lux and NtrC families’ clade. In the tree based on the muscle alignment, the RpfG clade is close to Lux–NtrC common clade, but shares a common well-supported node with the LytTR family (Fig. S12).

## Discussion

The QS mechanism includes a multitude of signal molecules and pathways, which aim to coordinate the activities of a bacterial population by changing gene expression according to the population density. The fact that this mechanism affects various processes makes this mechanism a potential target in antibiotics research. Few phylogenetic analyses on the bacterial QS exist, and given the multiple pathways contributing to QS, studies have so far focused only on the signalling molecules, or on specific pathways. Among them, the LuxI/R pathway of Gram-negative bacteria has been mainly studied. We chose to analyse homologous proteins of the QS pathways which belong to a widespread signal transduction system, the two component system (TCS). This mechanism includes pairs of a histidine kinase (HK) and its cognate response regulator (RR). The studied QS pathways are found in a wide variety of both Gram-positive and Gram-negative bacteria.

### Distributions of QS proteins

Looking at the distribution of the different QS pathways across bacterial taxa, the overall conclusion is that α- and γ-proteobacteria, clostridia and bacilli possess the highest number of QS proteins (Fig. 2). The most widespread proteins are QseB, DesA, FusR and LuxS (Fig. 3). These belong to various pathways and families and they have different roles, indicating no clear relationship between these characteristics and the number of bacterial groups containing them. The least widespread proteins are found in the two Com pathways, the Agr and the Lux pathway. These results indicate that the wide presence of one protein doesn’t mean a similar distribution for the rest of the proteins of the same pathway. This contradiction is indicated by the fact that the Lux pathway contains both some of the most widespread (LuxS) and some of the most limited protein types (LuxQ, LuxN, LuxU, LuxR). Also, there is a difference even in the distribution of the HK–RR pairs, i.e. between the HK and its cognate RR. This distribution may simply be the result of inadequacies in genome annotation, or it may be an indication of flexibility in the system, meaning that HKs can function with other than their ‘cognate’ RRs, in essence leading to mixed pathways. Although TCS pathways demonstrate a high specificity overall, such ‘cross-talk’ between different TCS pathways is possible [41]. In the case of ‘orphan’ HKs or RRs with no cognate protein [42, 43], an interaction between an orphan protein and a non-cognate one can also occur [43]. Also, given the QS map of KEGG (Fig. 4), QseC can interact with GlrR (QseF), which is not only a non-cognate RR, but it also belongs to a different family (NtrC); interestingly, QseC and GlrK both bind to adrenaline and noradrenaline. All of the interactions depicted in the QS maps of KEGG (maps ko02020 and ko02024) involve proteins of the OmpR family. QseB inhibits the cognate pair of FusK/FusR of the NarL family and CiaR inhibits ComC, ComD and ComE proteins of the LytTR family. Our findings about the distribution of the ComQXPASK pathway agree with an earlier suggestion about the possible presence of ComXQPA outside *Bacillus subtilis* species and other close relatives [44], as the full pathway (ComQXPA) is also present in clostridia, while chloroflexi contain the HK–RR pair.

Examining each protein family of our study separately, it seems that the most widespread HK–RR pairs of QS are those of the NarL, OmpR and NtrC families, whereas the Lux and LytTR are the least common, although the Lux pathway is the most well-known and well-studied QS pathway.

### Conserved domains

The HK–RR pairs belong to six families, as defined by KEGG: OmpR, LytTR, NarL, Lux, NtrC and ‘Other’. In both the OmpR and the LytTR family, the different protein types contain a small number of common conserved domains. On the other hand, HKs of the NarL (FusK) and Lux families show common domains and a number of variable domains; notably, the Lux family also includes hybrid HKs. The RRs of all families contain the response_reg domain and a domain with a HTH motif.

The Lux family consists exclusively of hybrid HKs, and all three of them activate a common phosphotransfer protein and RR. The NtrC family contains only the GlrK HK, which has the same conserved domains as the HKs of the OmpR family, a characteristic which is also evident in their close relationship on the phylogenetic tree. However, its cognate RR (GlrK) has various common domains with the RR of the Lux family. This is also reflected in the phylogenetic trees. Therefore, we conclude that similarities can be found not only among the protein types within a family, but also among different family members, which indicate a possible common evolutionary history.

There is partial agreement of our results with those of previous studies on the classification of HKs and RRs based on their conserved domains. The differences are mostly due to differences in the selection of proteins included in each study, and due to differences in classification based on domain structure alone versus phylogenetic analysis. For example, the HKs had been previously divided into 11 subfamilies based on multiple alignment of 348 HKs and comparison of the ‘homology boxes’, i.e. groups of highly conserved amino acids, which are assumed to be important in substrate binding, catalysis or the structure of the protein: [45]. In the that study, ComP was classified in group HPK7, CiaH in HPK1a, NisK in HPK3c and, RpfC in HPK1b [45]. However, only some of the HKs mentioned here were also included in that study, and this way of classification differs from the KEGG database and our phylogenetic tree results, as CiaH and NisK (OmpR family members) belong to separate groups and RpfC (hybrid HK) is placed with the hybrid HKs of the NarL family (although hybrid HKs were placed in other HPK groups as well). Capra and Laub classified the HKs into four groups depending on the presence or absence of period circadian protein–aryl hydrocarbon receptor nuclear translocator protein–single-minded protein (PAS), histidine kinases–adenylate cyclases–methyl accepting proteins and phosphatases (HAMP) and cGMP-specific phosphodiesterases–adenylyl cyclases and formate hydrogenlyase transcriptional activator (GAF) domains [21]. According to the authors, most HKs have at least one domain between the transmembrane part of the protein and the histidine phosphotransfer domain, with PAS, HAMP and GAF being ‘by far the most common’ ones. However, our studied proteins had no PAS, HAMP and GAF domains, with the exception of QseC and QseE–GlrK, in which the HAMP domain had an E-value in KEGG and SMART which didn’t pass out threshold to be included in the conserved domains of our study.

The DNA-binding domains (effector domains) of RRs have been classified into three subfamilies: OmpR, Fix J and NtrC [46], or more recently into five groups [21]. The classification of the RRs according to Hakenbeck and Stock [46] is the same as the data of KEGG and our study. CiaR and NisR were classified as members of the OmpR family and ComA was classified as a member of the FixJ family. The effector domains for the classification of the RRs were different in the research of Capra and Laub, as no GGDEF and methyltransfer domains were present in the proteins of our study. However, the LuxO and GlrR proteins possessing the AAA_5 domain were grouped close to each other in our phylogenetic trees, complying with the classification criterion of Capra and Laub. By comparing the receiver domains, the RRs have been classified into eight subfamilies (R_A_–R_H_) [45], although it has been proposed that the comparison of the RRs for their classification should be based only on the effector domain, due to the fact that the receiver domains of different RRs are highly conserved and therefore not very informative for classification [47]. The results of Grebe and Stock [45] are similar to ours: CiaR and NisR were classified to the R_A1_ group and ComA to R_E_, while AgrA and ComE are grouped to R_D_, both of which belong to the LytTR family in our study.

### Phylogenetic trees

In all the inferred phylogenetic trees of each family, the sequences of both HKs and RRs are grouped in distinct clades according to their protein type and in many cases, with high bootstrap values. The same happens in the phylogenetic trees of all families together, with distinct clades for sequences of different family and protein types. Also, the relationship among protein types (either HKs or RRs) is generally the same in the tree of each family and of all families together. In most cases, the relations among the HKs are also reflected by the relations of their cognate RRs, indicating a common evolutionary history, as has already been suggested during previous studies on other proteins of the TCS [28].

For the OmpR family, the clades of QseC and CiaH share a common node in the tree of all HK families together, and the pattern is recapitulated for their cognate RRs, CiaR and QseB. The OmpR clades of the HKs are closer to the NtrC family in the tree of all HK families together, but their cognate RRs don’t show this relationship. Regarding the Lux HKs, the clades of CqsS and LuxN share a common node in the tree of all HK families together. The LuxQ sequences group with RpfC sequences. Their common RR (LuxO) is in the same clade either with RpfG (MAFFT) or with QseF/GlrR (muscle) in the tree of all RRs. The protein members of the LytTR family have separate clades for the AgrC–ComD and AgrA–ComE sequences in all trees. The AgrC2 and AgrA2 sequences belong to the AgrC and AgrA clade, which is confirmed by all the inferred phylogenetic trees. The position of the RpfC–RpfG and GlrK–GlrR sequences (NtrC family) is slightly different for the HKs and their RRs. The RpfC sequences are closer to LuxQ in the tree of all HK families together, whereas RpfG is closer to the pair LuxO–GlrR. GlrK is closer to the OmpR family but GlrR is in the same clade with LuxO.

The results of a comparison of the phylogenetic trees of all HKs and all RRs indicate two clusters of the QS protein families: (1) Lux with RpfG, and (2) NarL with LytTR, as well as clusters of protein types per family as outlined above. However, OmpR and NtrC demonstrate a different relationship, comparing the trees for the HKs and RRs.

These evolutionary relationships highlight a common evolutionary history, and can inform future applications, such as the design of novel inhibitors for pathogenic QS systems. In fact, there have already been several scientific papers about quorum quenching for some of the proteins of our study. Diol-containing compounds, boronic acids and sulphones have been suggested as antagonists of the signalling molecules which bind with LuxP and as potential solutions to biofilm formation [48]. Other molecules inhibiting the QS pathways of Gram-negative pathogens have been reported, including for the HKs and RRs of the Lux family and RpfC–RpfG, by blocking the receptors, inhibiting the production of the signalling molecules, or by degrading them [49]. The inhibition of the Agr pathway of *Staphylococcus aureus* has also been studied, with natural and synthetic substances: the cyclodepsipeptide Solonamide B of *Photobacterium halotolerans* interferes with the binding of AIPs to AgrC, whereas ω-hydroxyanodin from *Penicillium restrictum* binds to AgrA and therefore inhibits the expression of the regulated genes [50]. Savirin (a *Staphylococcus aureus* virulence inhibitor) is another small molecule that selectively targets AgrA [51]. The synthetic small molecule inhibitor LED209 is selective to QseC [52]. The genes *comD* (HK) and *comE* (RR) have also been found to be downregulated by a small molecule containing a 2-aminoimidazole subunit (2A4) along with five other biofilm-related genes of *Streptococcus mutans* [53]. The antibiotic mupirocin when used in sub-inhibitory concentrations on high-level mupirocin-resistant, methicillin-resistant *Staphylococcus aureus* (MRSA) strains interferes with the *agrA* genes [54]. Our prediction is that such inhibitors are more likely to also interfere with protein types similar to the original target protein, as highlighted by the phylogenetic analysis, than with more distant ones, e.g. an inhibitor for a Lux family HK may also affect NtrC family HKs. On the other hand, pathway cross-talk in species with multiple QS systems may hinder these inhibitory effects. Broader inhibition approaches have also been suggested, with the ATP-binding domain of HKs (HATPase domain) being the main target for TCS pathways inhibition [55, 56].

Two-component signal transduction arose early in bacterial evolution after their separation from the last common ancestor, with significant diversification throughout the subsequent bacterial speciation; HKs arose from ATPases of the GHKL superfamily and pyruvate dehydrogenase kinases (PDKs), while the origin of the RRs remains unclear [21, 28]. Regarding the origin of two-component quorum sensing systems, the results of a phylogenetic analysis for the proteins LuxI, LuxR and LuxS in firmicutes, proteobacteria and actinobacteria, indicated that these proteins appeared very early in the evolutionary path of the bacteria, but with multiple occasions of horizontal gene transfer resulting in their present-day distribution [15]. In a study of the ComQXPA pathway in firmicutes, the phylogenetic tree of HK proteins clustered all the ComP proteins from various organisms together, instead of gathering them with other HKs of the same organism, indicating an early origin of ComP [29]. The phylogenetic analyses presented here are unrooted and assume an early origin of these pathways in bacterial evolution. We present the distribution and evolutionary relationships of different HK and RR protein types and families, but a larger-scale analysis of all available sequences for two-component QS systems and other HK–RR pairs would be needed to confidently address the issue of the root of quorum sensing.

## Conclusions

In this study we examine the distribution of different bacterial QS pathways across taxa, as well as the domain structure and evolutionary relationships of the pathways' core proteins. We have chosen to focus on QS systems which conform to the structure of TCSs, and particularly on the HKs and RRs, which are a common feature of all TCS pathways. The HK–RR pairs belong to six families, as defined by KEGG: NarL, OmpR, Lux, LytTR, NtrC and ‘Other’. The distribution of the different QS systems across taxa, indicates that proteobacteria, clostridia and bacilli possess the highest number of QS proteins. However, many species encode only certain proteins of each pathway, and there are differences even in the distribution of an HK and its cognate RR. This indicates a flexibility in the system and possible ‘cross-talk’ between different pathways. In terms of protein families, it seems that the most widespread HK–RR pairs are those of the NarL, OmpR and NtrC families, whereas the Lux and LytTR are the least common; since the Lux pathway is the most well-studied QS pathway, this indicates a gap in our knowledge of the workings of the most widespread QS pathways. In terms of conserved domains, similarities can be found not only among the protein types within a family, but also among different family members (e.g. between the OmpR and the LytTR family HKs, or between the NtrC and OmpR HKs). In the phylogenetic analyses, we see distinct clades for sequences of different family and protein types, while the relationship among protein types is generally the same in the trees of each family and of all families together. In most cases, the relations among the HKs are also reflected by the relations of their cognate RRs, indicating a common evolutionary history. The evolutionary relationships of the different QS pathways deserve further study as they can have an effect on the applicability of knowledge gleaned from one pathway (e.g. Lux) to other QS pathways.

## Supplementary Data

Supplementary material 1
