# Supplementary material for: Evolution of two-component quorum sensing systems
Source: Access Microbiol. 2022 Jan 12;4(1):000303. doi: 10.1099/acmi.0.000303 (PMC8895600; doi:10.1099/acmi.0.000303)
Supplement: Supplementary material 1 [file acmi-4-0303-s001.pdf]

Supplementary Figures and Supplementary Tables

Supplementary Figure S1A: Classification of the HK-RR cognate pairs in the KEGG database (KEGG map: ko2020). The chosen proteins are indicated in red frames.

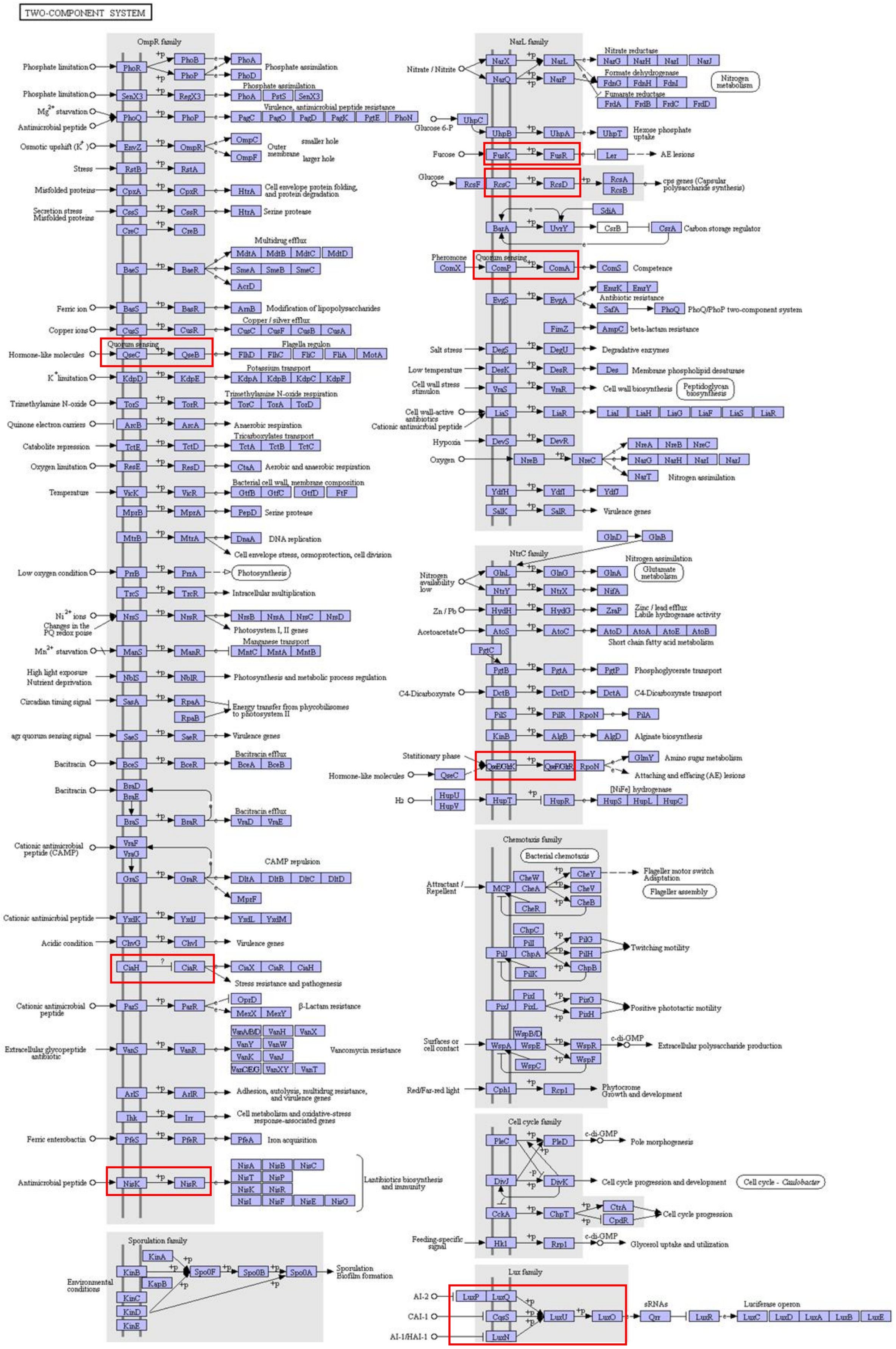

Supplementary Figure S1B: Classification of the HK-RR cognate pairs in the KEGG database (KEGG map: ko2020). The chosen proteins are indicated in red frames.

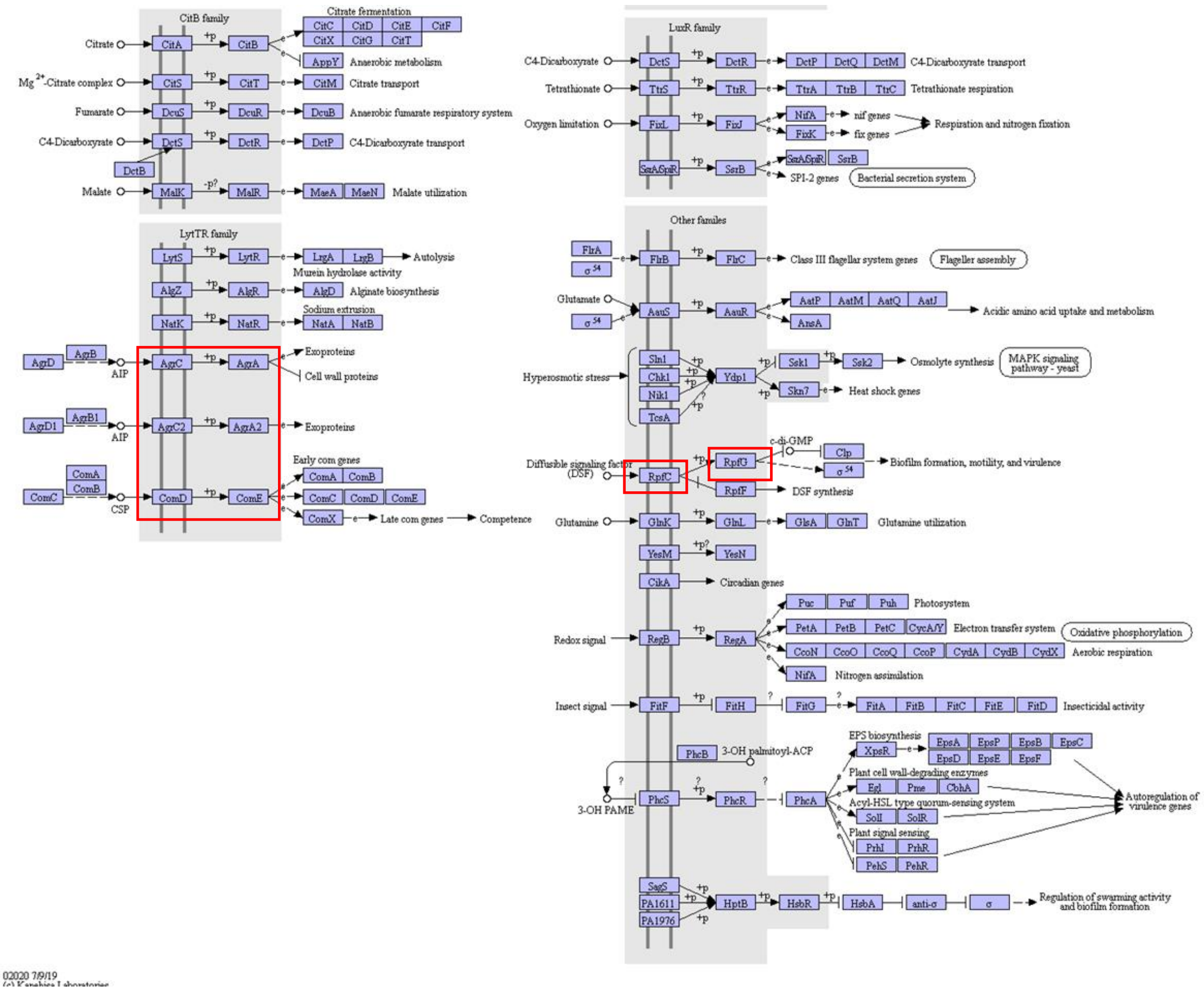

Supplementary Figure S2A: Quorum Sensing map in the KEGG database (KEGG map: ko2024). The chosen proteins are indicated in red frames.

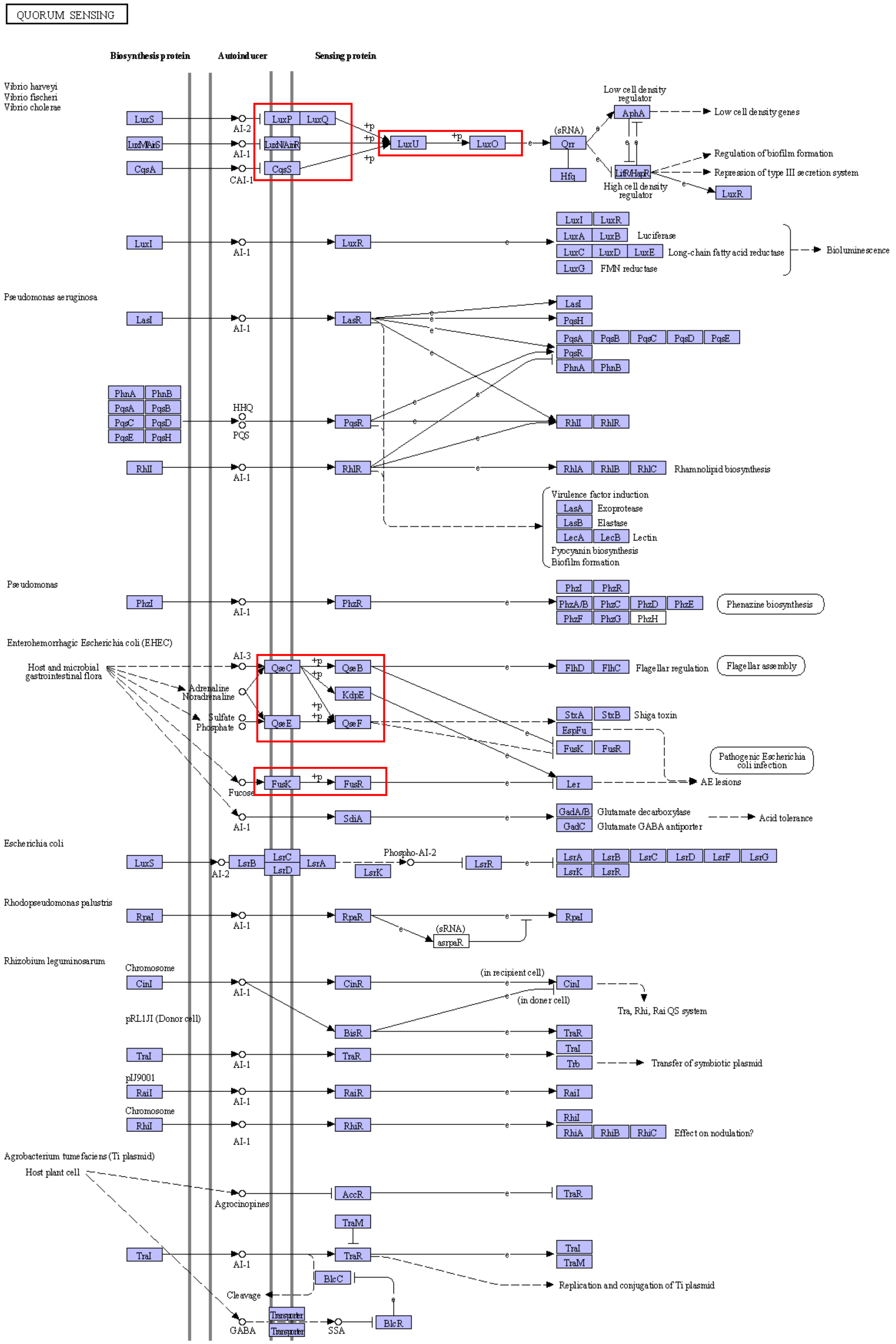

Supplementary Figure S2B: Quorum Sensing map in the KEGG database (KEGG map: ko2024). The chosen proteins are indicated in red frames.

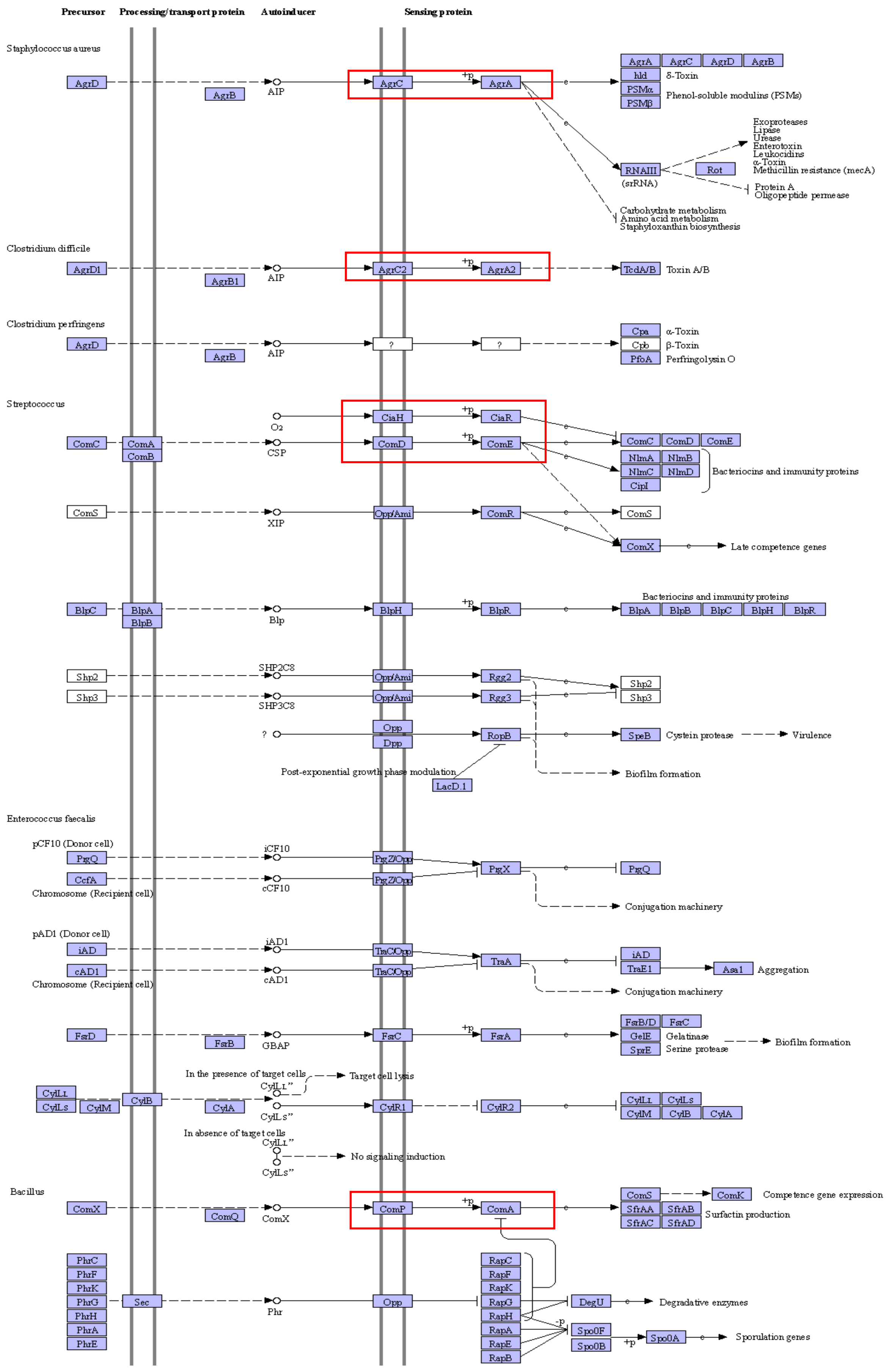

Supplementary Figure S2C: Quorum Sensing map in the KEGG database (KEGG map: ko2024. The chosen proteins are indicated in red frames.

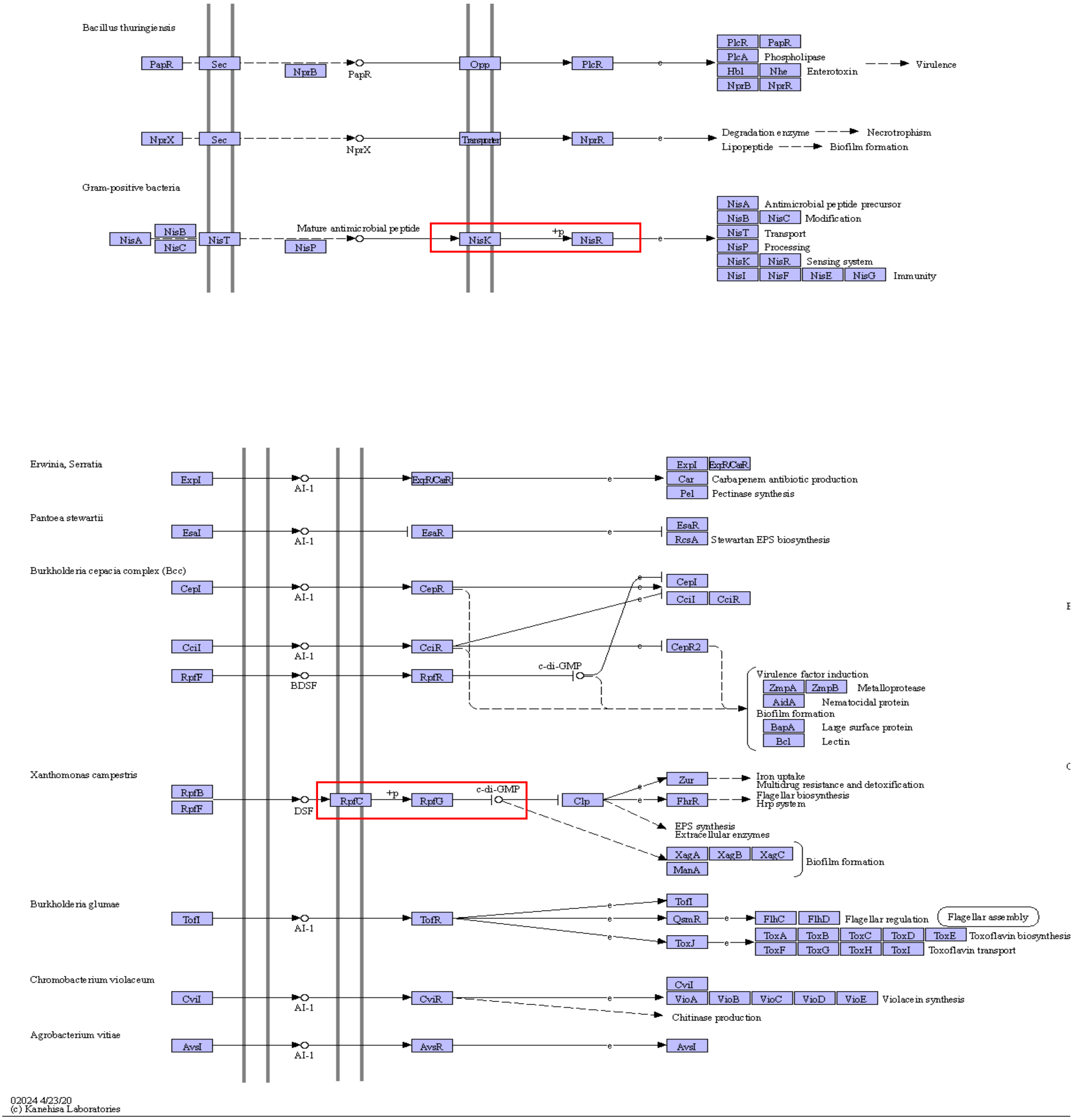

Supplementary Figure S3A: MAFFT alignment of all the HKs

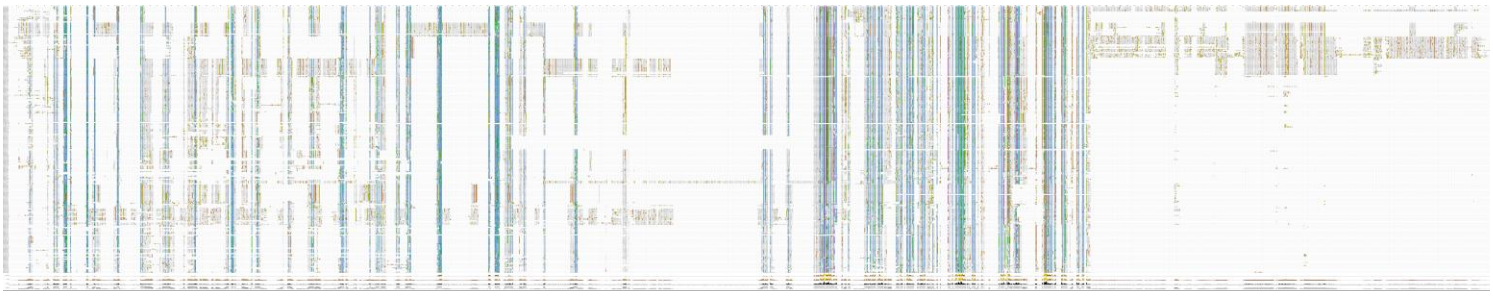

Supplementary Figure S3B: The parts of the alignment in grey were removed using Mesquite.

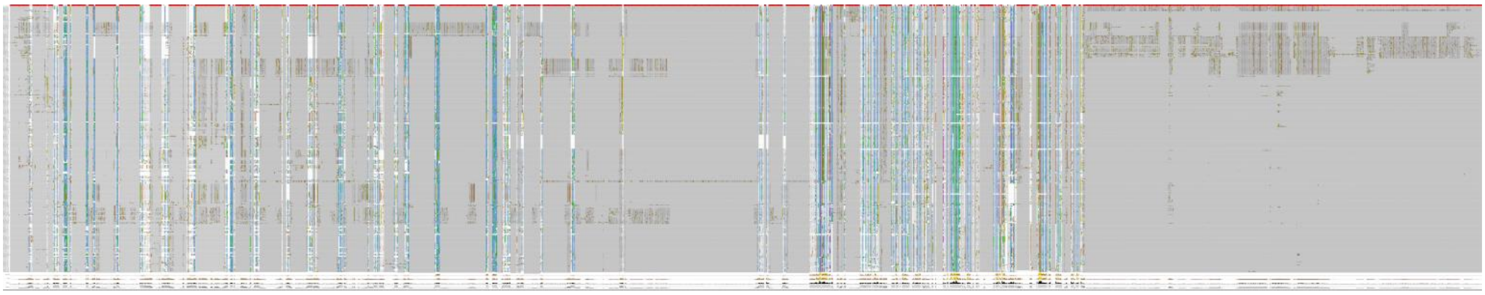

Supplementary Figure S3C: The final part of the MAFFT HKs alignment that remained after the trimming.

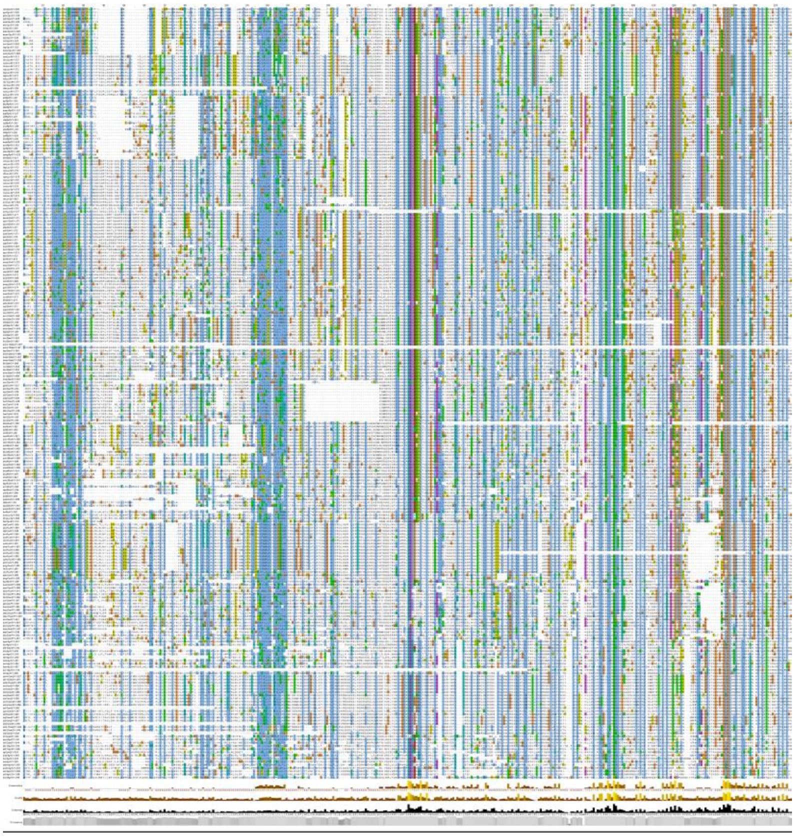

Supplementary Figure S4A: MUSCLE alignment of all the HKs

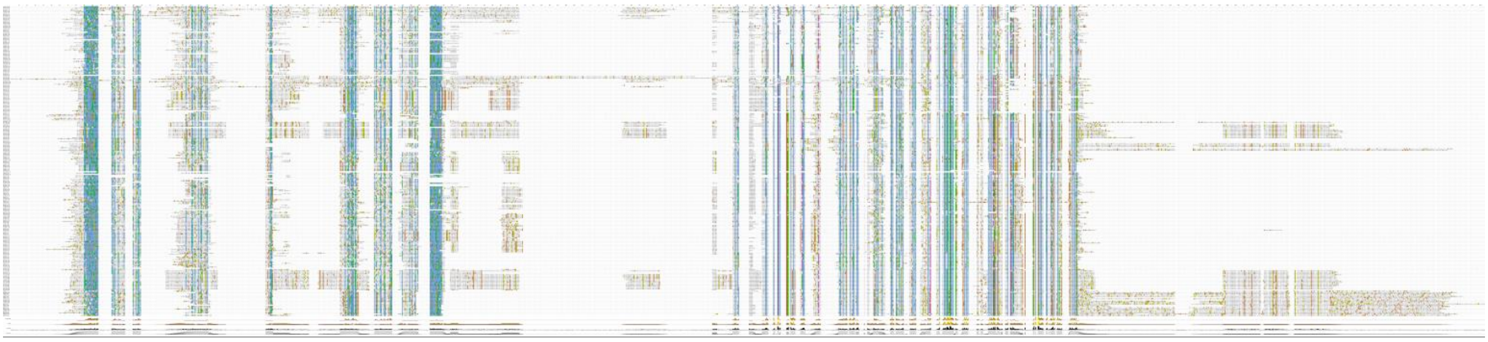

Supplementary Figure S4B: The parts of the alignment in grey were removed using Mesquite.

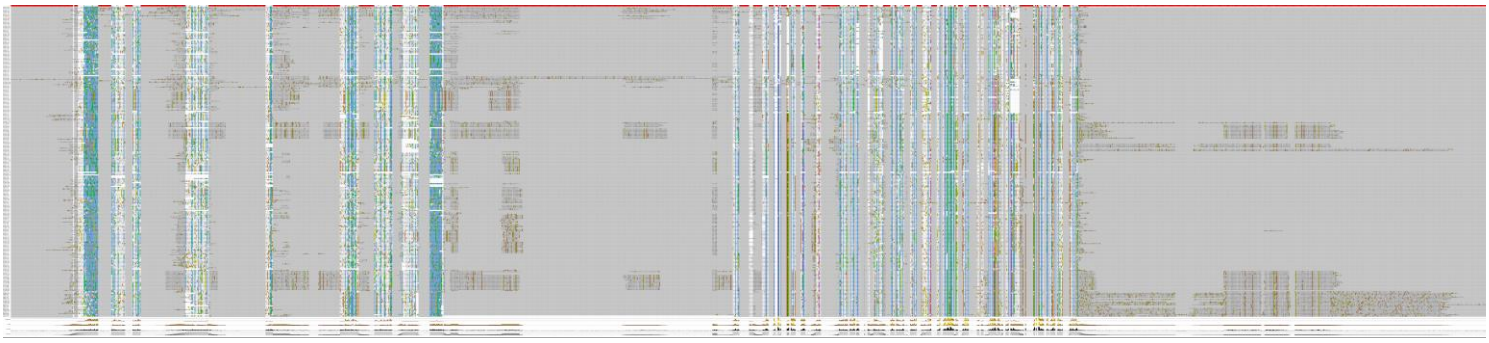

Supplementary Figure S4C: The final part of the MUSCLE HKs alignment that remained after the trimming.

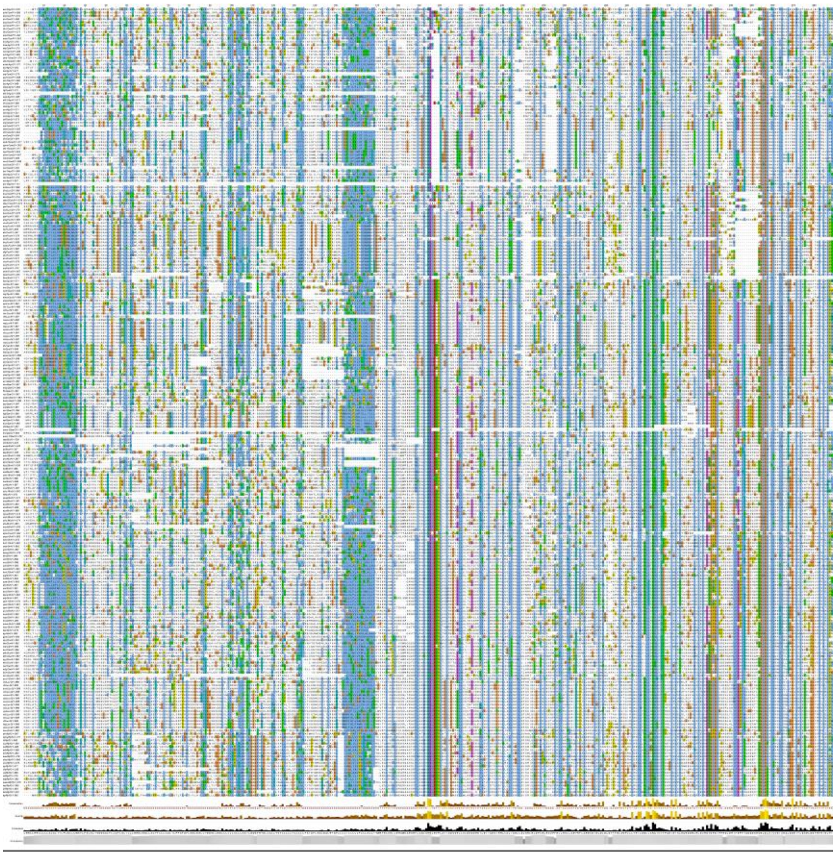

Supplementary Figure S5A: The MAFFT alignment of all the RRs.

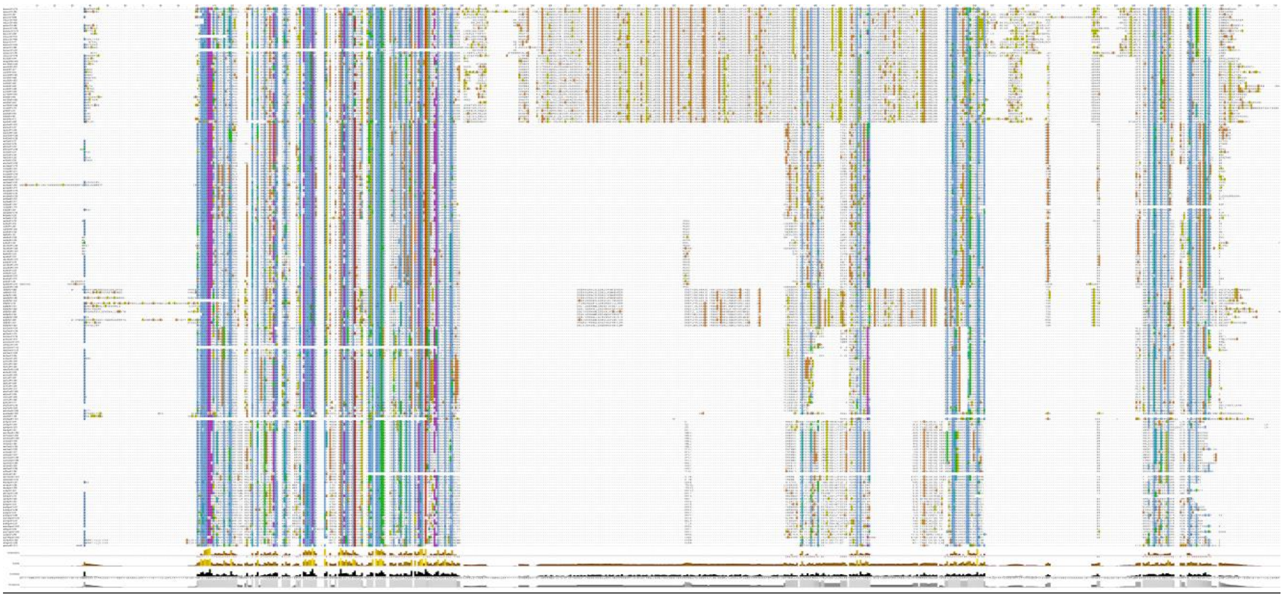

Supplementary Figure S5B: The parts of the alignment in grey were removed using Mesquite.

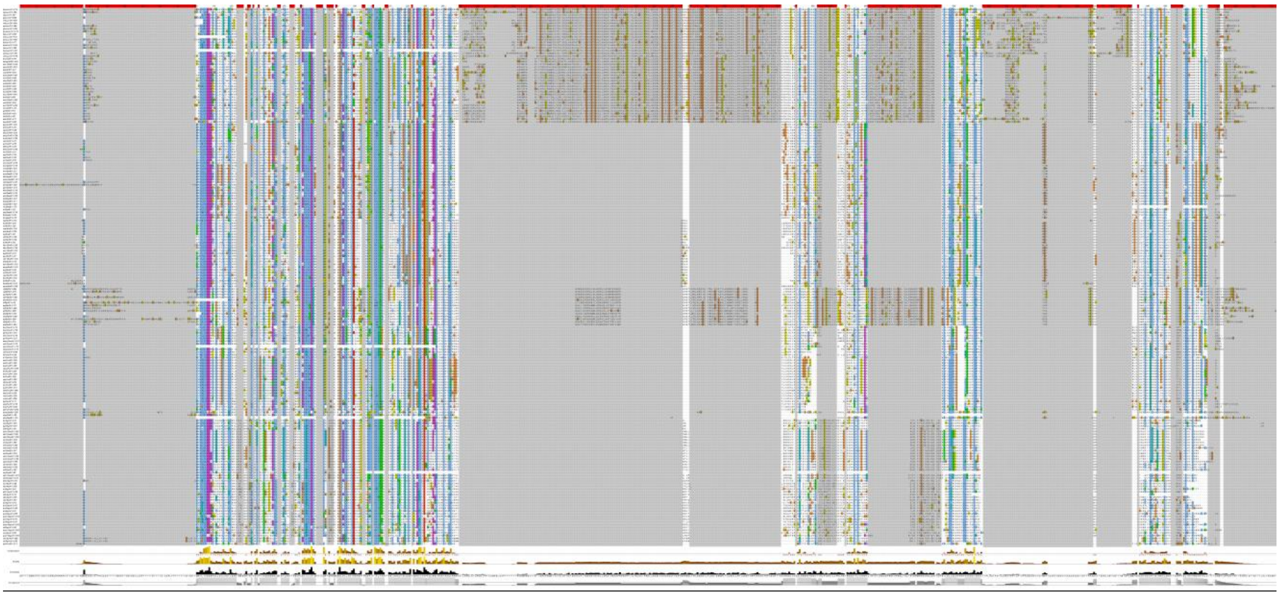

Supplementary Figure S5C: The final part of the MAFFT RRs alignment that remained after the trimming.

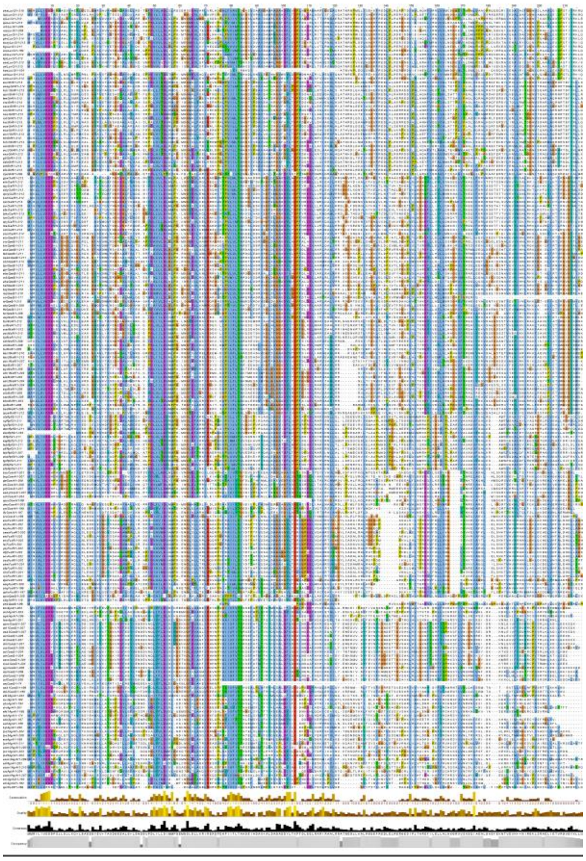

Supplementary Figure S6A: MUSCLE alignment of all the RRs.

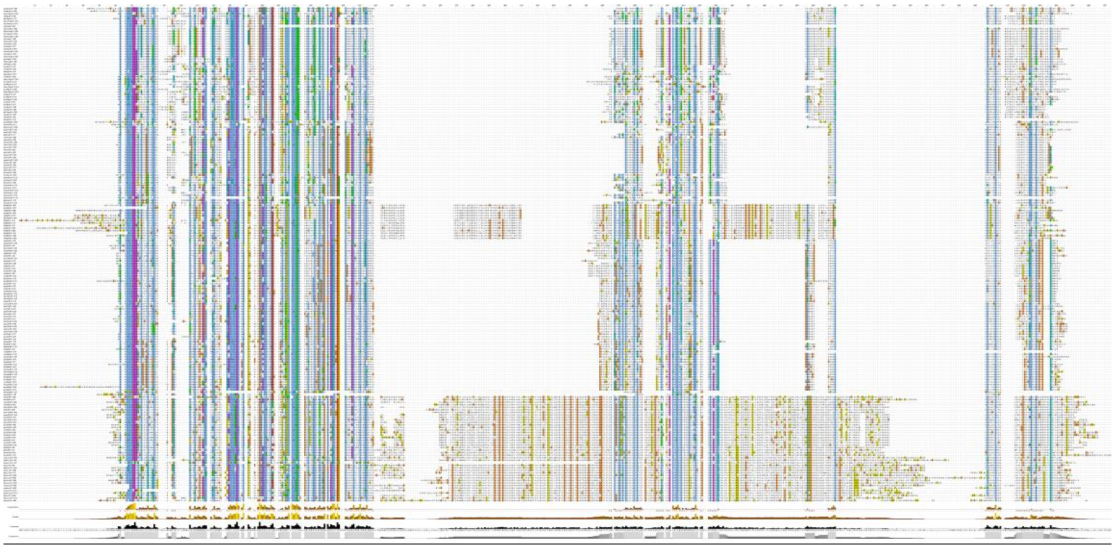

Supplementary Figure S6B: The parts of the alignment in grey were removed using Mesquite.

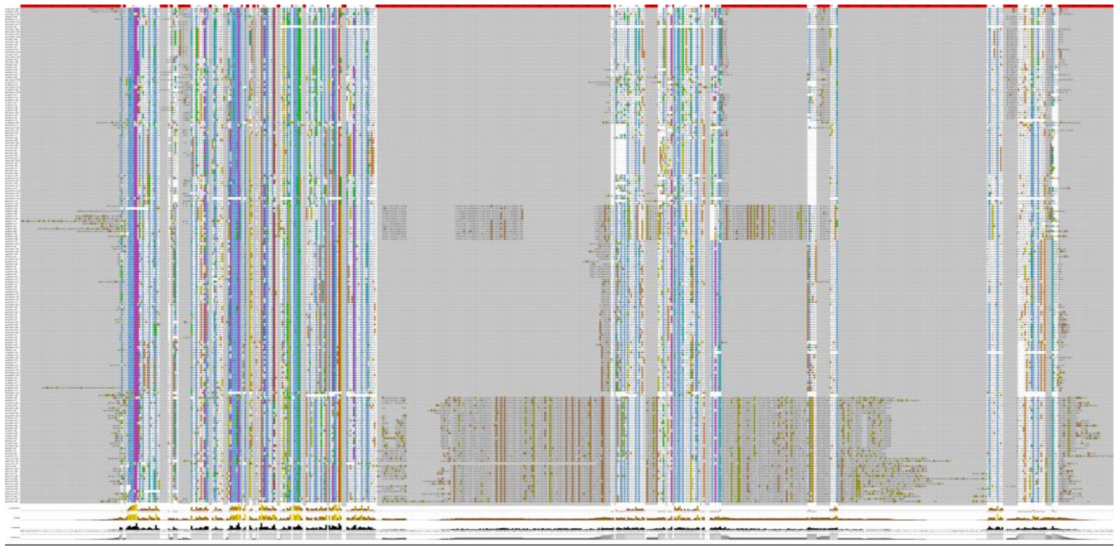

Supplementary Figure S6C: The final part of the MUSCLE RRs alignment that remained after the trimming.

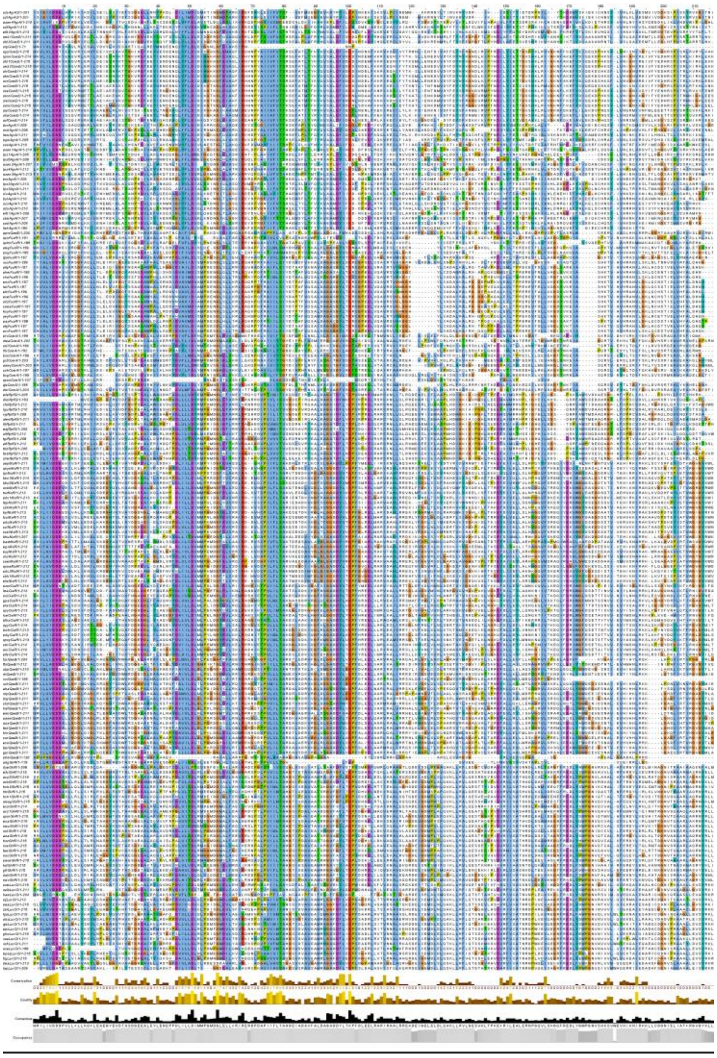

Supplementary Figure S7: Phylogenetic analysis of the NarL family. Both trees reconstructed using PhyML based on the MUSCLE alignment. (A) Histidine Kinases. (B) Response Regulators. The color of the bullets indicates the bootstrap value of the nodes. White: 50% -80%, Grey: 80%-95%, Black: over 95%

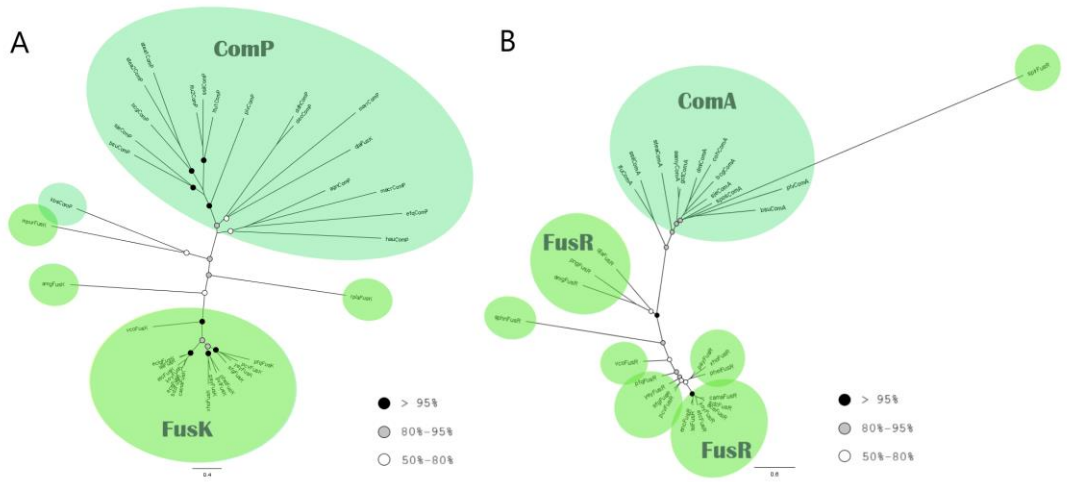

Supplementary Figure S8: Phylogenetic analysis of the OmpR family. Both trees reconstructed using PhyML based on the MUSCLE alignment. (A) Histidine Kinases. (B) Response Regulators.

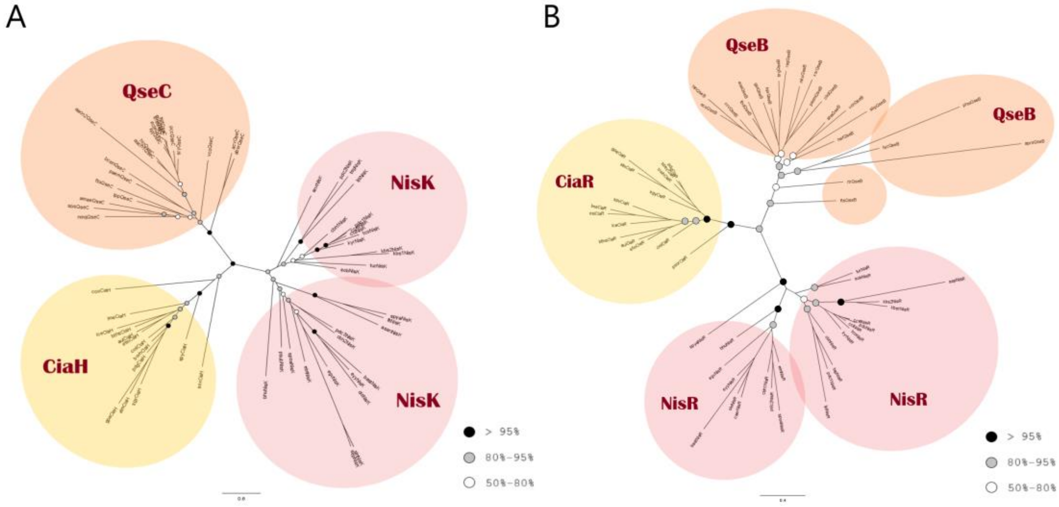

Supplementary Figure S9: Phylogenetic analysis of the Lux family histidine kinases. Tree reconstructed using PhyML based on the MUSCLE alignment.

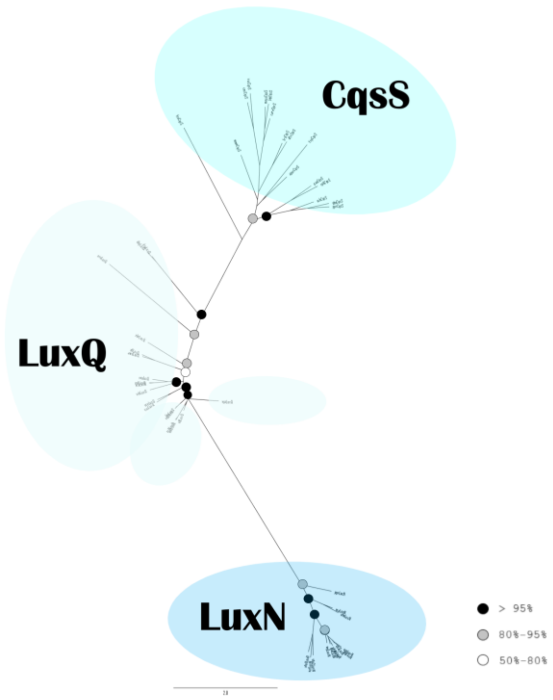

Supplementary Figure S10: Phylogenetic analysis of the LytR family. (A) Histidine Kinases. (B) Response Regulators. Both trees reconstructed using PhyML based on the MUSCLE alignment.

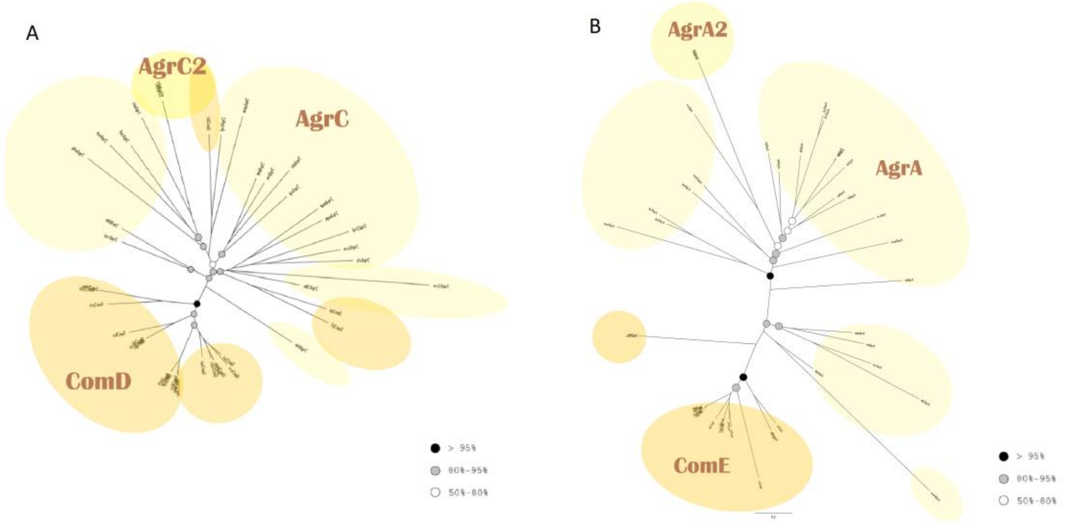

Supplementary Figure S11: Phylogenetic analysis of all histidine kinase families. Tree reconstructed using RaxML based on the MUSCLE alignment.

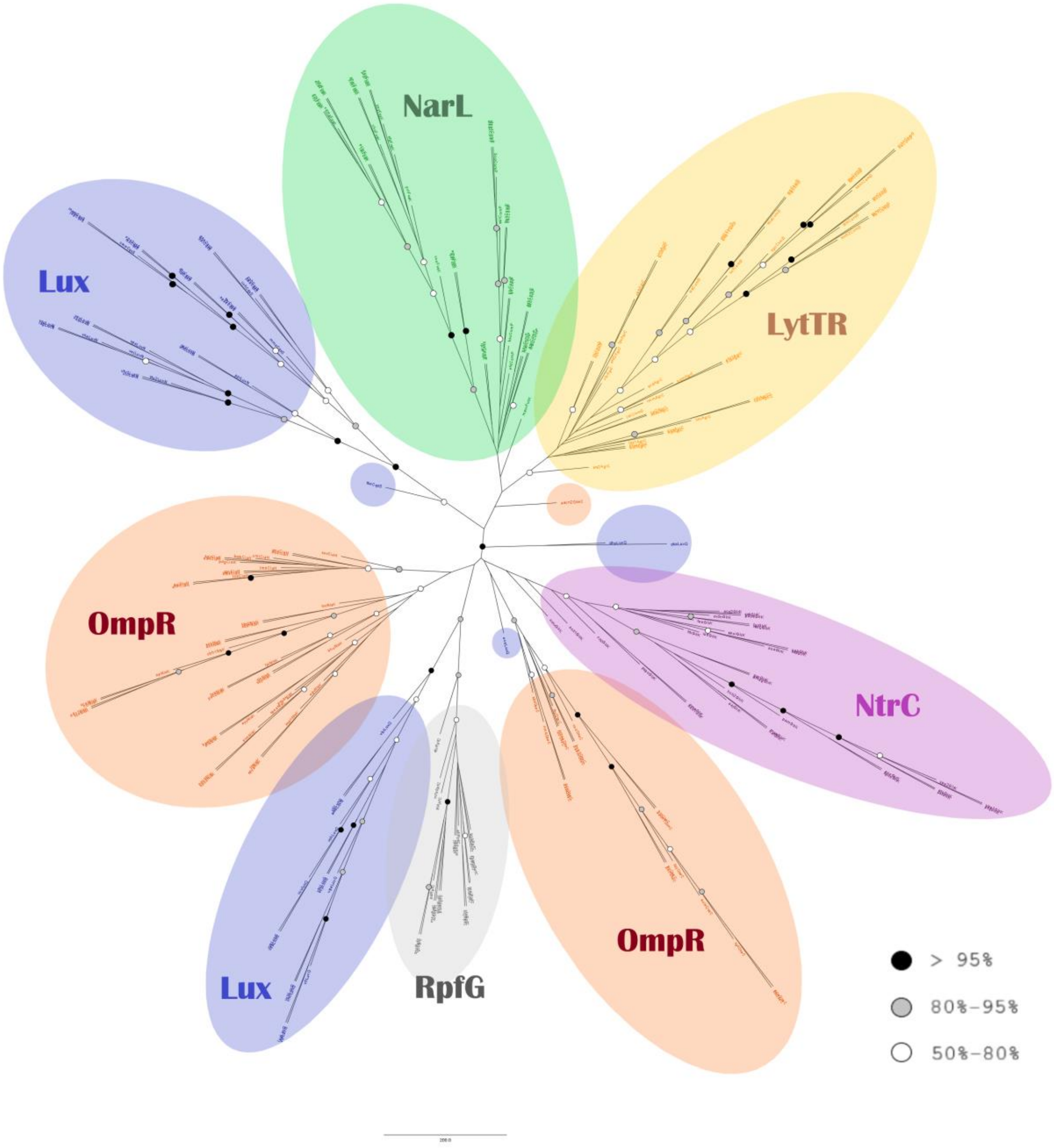

Supplementary Figure S12: Phylogenetic analysis of all response regulator families. Tree reconstructed using RaxML based on the MUSCLE alignment

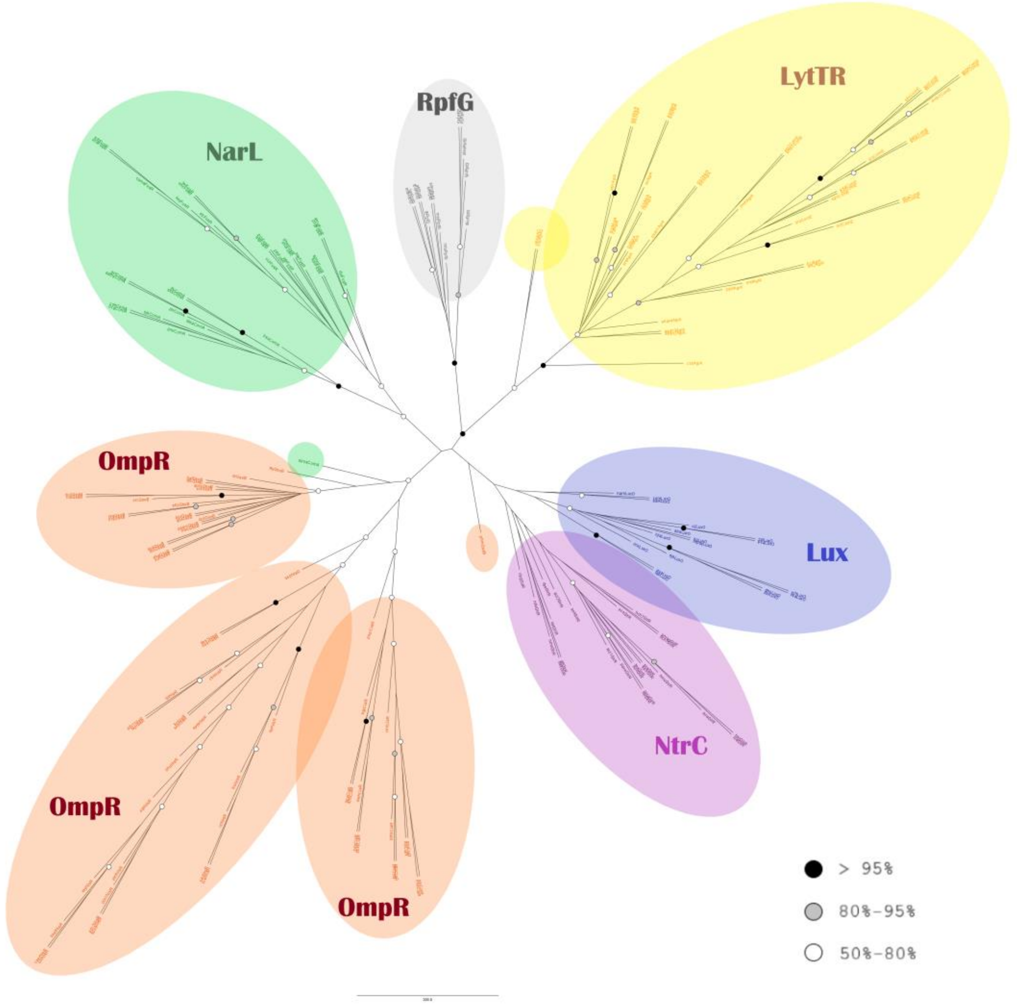

Supplementary Table S1: The presence of the proteins in several QS pathways was examined in various bacterial classsifications.

Phylum	Class	Family	Genus
gemmatimonadetes	a-proteobacteria	aquificae	leptospira (spirocahetes)
acidobacteria	b-proteobacteria		deinococci
chlorobi	g-proteobacteria		nitrospira
bacteroidetes	d-proteobacteria		chlamydiae
verrucomicrobia	e-proteobacteria		borrelia (spirochaetes)
chloroflexi	clostridia (firmicutes)		
cyanobacteria	bacilli (firmicutes)		
actinobacteria			
fusobacteria			
mollicutes (tenericutes)			
dictyoglomi			
thermotogae			
elusimicrobia			
Planctomycetes			
Fibrobacteres			
Chrysiogenetes			
Deferribacteres			
Other Spirochaetes			

Supplementary Table S2: PFAM and InterPro entries for each domain discussed in this study, as well as the entry of the superfamily (NCBI-CDD) and Pfam clan they belong to.

DOMAINS	**PFAM entry**	**InterPro entry**	**Superfamily (NCBI-CDD)**	**pfam clan**
LuxQ-periplasm	PF09308	IPR015387	cl07810	CL0165
Response_reg	PF00072	IPR001789	cl19078	CL0304
HisKA	PF00512	IPR003661	cl29579	CL0025
HisKA_3	PF07730	IPR011712	cl38245	
HATPase_c	PF02518	IPR003594	cl00075	
HATPase_c_2	PF13581	IPR003594		
HATPase_c_5	PF14501	IPR032834		
Hpt	PF01627	IPR008207	cl00086	…
2CSK_N	PF08521	IPR013727	cl07224	CL0165
Peripla_BP_4	PF13407	IPR025997	cl10011	CL0144
Sigma54_activat	PF0158	IPR002078	cl37971	CL0023
Sigma54_activ_2	PF14532	IPR002078	cl37881	
Sigma54_AID	PF00309	IPR000394	cl08259	…
Sigma54_DBD	PF04552	IPR007634	cl10632	
Sigma54_CBD	PF04963	IPR007046	cl29309	
Sigma70_r4_2	PF08281	IPR013249	cl22867	CL0123
Sigma70_r4	PF04545	IPR007630		
Trans_reg_C	PF00486	IPR001867	cl21459	
AAA_5	PF07728	IPR011704	cl38244	CL0023
HTH_8	PF02954	IPR002197	cl22895	
GerE	PF00196	IPR000792	cl21459	
HD	PF01966	IPR006674	cl21469	CL0237
HD_5	PF13487	…		
ECH_1	PF00378	IPR001753	cl23717	CL0127
ECH_2	PF16113	IPR032259		
MASE1	PF05231	IPR007895	…	…
LytTR	PF04397	IPR007492	cl04498	CL0049

Supplementary Table S3A: The number of HK amino acid sequences which were used for the phylogenetic trees, and the percentage representation of taxa, families and species out of the total available ones in the KEGG database are indicated.

	NarL family			OmpR family			Lux family			LytTR family		NtrC family			Other		
	Taxa	Families	Total	Taxa	Families	Total	Taxa	Families	Total	Families	Total	Taxa	Families	Total	Taxa	Families	Total
	ComP			QseC			LuxQ			AgrC		GlrK (QseE)			RpfC		
KEGG	5	9	159	28	68	1190	3	3	59	24	714	19	44	652	13	19	99
Our study	5	9	17	9	14	20	3	3	17	15	20	16	29	33	13	19	20
%	100%	100%	11%	32%	21%	2%	100%	100%	29%	63%	3%	84%	66%	5%	100%	100%	20%
	FusK			CiaH			LuxN			AgrC2							
KEGG	7	12	144	5	13	256	1	1	35	1	2						
Our study	12	12	20	5	13	14	1	1	13	1	2						
%	86%	100%	14%	100%	100%	5%	100%	100%	37%	100%	100%						
				NisK			CqsS			ComD							
KEGG				11	23	367	7	9	100%	2	52						
Our study				11	21	29	7	9	100%	2	22						
%				100%	91%	8%	100%	100%	21%	100%	42%						

Supplementary Table S3B: The number of RR amino acid sequences which were used for the phylogenetic trees, and the percentage representation of taxa, families and species out of the total available ones in the KEGG database are indicated.

	NarL family			OmpR family			Lux family			LytTR family			NtrC family			Other		
	Taxa	*Families*	*Total*	*Taxa*	*Families*	*Total*	*Taxa*	*Families*	*Total*	*Taxa*	*Families*	*Total*	*Taxa*	*Families*	*Total*	*Taxa*	*Families*	*Total*
	ComA			QseB			LuxO			AgrA			GlrR (QseF)			RpfG		
KEGG	2	4	150	36	73	1386	11	14	129	6	19	623	25	53	683	7	11	83
Our study	2	4	10	23	23	23	11	14	18	6	19	21	24	21	24	7	11	15
%	100%	100%	7%	64%	32%	2%	100 %	100%	14%	100%	100%	3%	96%	40%	4%	100 %	100%	18%
	FusR			CiaR						AgrA2								
KEGG	6	12	193	4	14	261				1	2	2						
Our study	6	12	20	4	14	15				1	2	2						
%	100%	100%	10%	100%	100%	6%				100%	100%	100%						
				NisR						ComE								
KEGG				10	20	377				1	1	49						
Our study				10	20	25				1	1	1						
%				100%	100%	7%				100%	100%	2%						
